# Segmentation of Image Data from Complex Organotypic 3D Models of Cancer Tissues with Markov Random Fields

**DOI:** 10.1371/journal.pone.0143798

**Published:** 2015-12-02

**Authors:** Sean Robinson, Laurent Guyon, Jaakko Nevalainen, Mervi Toriseva, Malin Åkerfelt, Matthias Nees

**Affiliations:** 1 Department of Mathematics and Statistics, University of Turku, Turku, Finland; 2 Industrial Biotechnology, VTT Technical Research Centre of Finland, Turku, Finland; 3 Université Grenoble-Alpes, F-38000 Grenoble, France; 4 CEA, iRTSV, Biologie à Grande Echelle, F-38054 Grenoble, France; 5 INSERM, U1038, F-38054 Grenoble, France; 6 School of Health Sciences, University of Tampere, Tampere, Finland; 7 Institute of Biomedicine, University of Turku, Turku, Finland; 8 Turku Centre for Biotechnology, University of Turku, Turku, Finland; Ghent University, BELGIUM

## Abstract

Organotypic, three dimensional (3D) cell culture models of epithelial tumour types such as prostate cancer recapitulate key aspects of the architecture and histology of solid cancers. Morphometric analysis of multicellular 3D organoids is particularly important when additional components such as the extracellular matrix and tumour microenvironment are included in the model. The complexity of such models has so far limited their successful implementation. There is a great need for automatic, accurate and robust image segmentation tools to facilitate the analysis of such biologically relevant 3D cell culture models. We present a segmentation method based on Markov random fields (MRFs) and illustrate our method using 3D stack image data from an organotypic 3D model of prostate cancer cells co-cultured with cancer-associated fibroblasts (CAFs). The 3D segmentation output suggests that these cell types are in physical contact with each other within the model, which has important implications for tumour biology. Segmentation performance is quantified using ground truth labels and we show how each step of our method increases segmentation accuracy. We provide the ground truth labels along with the image data and code. Using independent image data we show that our segmentation method is also more generally applicable to other types of cellular microscopy and not only limited to fluorescence microscopy.

## Introduction

Cellular processes naturally occur in three dimensions (3D) and cells are typically embedded in extracellular matrix, which is a key component of the cellular microenvironment. Accordingly, physiologically relevant cell culture models are increasingly designed in 3D formats embedded in extracellular matrix to capture complex tissue-like biology more faithfully [1]. A solid tumour represents a disturbed and complex tissue with its own characteristic tissue homeostasis and surrounding tumour microenvironment. Tumour cell plasticity and important properties such as differentiation versus tumour cell invasion are strongly influenced by the tumour microenvironment. To recapitulate the features of solid tumours *in vitro* requires cell based assays that simultaneously mimic the extracellular matrix and tumour microenvironment, homeotypic and heterotypic cell-cell contacts, and allow the formation of relevant cell-matrix interactions. Organotypic 3D cell culture techniques currently represent the most biologically relevant *in vitro* models for the investigation of epithelial cancer differentiation, polarisation and invasion [1–3]. However the lack of appropriate microscopy image analysis methods has so far limited the successful implementation and interpretation of such models.

Apart from cancer cells, different stromal cells represent the most critical component of the tumour microenvironment. Cancer-associated fibroblasts (CAFs) are the most abundant stromal cell type in most carcinomas and play an essential role in tumour progression [4–6]. CAFs therefore represent an important target for cancer therapies. The interaction between tumour cells and CAFs is still poorly understood and more reliable and robust cell culture models that better recapitulate the complex histology of *in vivo* tumours are required to study tumour-stroma interactions.

We consider multichannel 3D stack image data from a complex organotypic 3D cell culture model of prostate cancer tumour cells co-cultured with CAFs. Our 3D cell culture model and imaging protocol relates to the multicellular level in which the overall attributes of tumour organoids and CAF structures are more critical than capturing individual cells or cell nuclei. Acquired with a relatively large distance between images in the stack, the resolution of the image data within an image was up to 20 times the resolution between images in the stack.

Healthy prostate tissues are formed of acini, which are hollow clusters of cells that form the smallest functional units of a secretory gland. In early stage prostate cancer these acini start to fill up with pre-malignant or malignant cells to become solid spheroids. The shape and size of multicellular organoids contains valuable morphometric information [7] and our interest lies in the multicellular tumour and CAF structures as distinct informative objects. Accurate segmentation of both the multicellular tumour and CAF structures is the first step in investigating in which format and scale these cell types may be forming direct cell-cell contacts. Hence our aim is to produce an automatic segmentation for both types of objects without assuming prior shape information or considering individual cells.

Automated computer vision typically allows for much greater efficiency and objectivity than manual human analysis [8]. Segmentation and the identification of portions of the image that are of interest is the first step in many computer vision applications. Simple thresholding methods form the basis of many typical segmentation methodologies [9]. Popular freeware image analysis platforms such as Cell Profiler [10] and ImageJ/Fiji [11] provide practical implementations of such segmentation methodologies specifically designed for cellular microscopy data and allow for analysis beyond segmentation in specialised pipelines for particular data sets. However such analysis is typically focused on high resolution microscopy data at single cell or even sub-cellular levels and is also mainly focussed on two dimensional (2D) monolayer cell cultures, although 3D volume viewing and rendering is possible in ImageJ. Commercial software products that focus on 3D cellular microscopy image analysis such as Imaris (Bitplane) and Volocity (PerkinElmer) are designed for very detailed analysis of high resolution images at the single cell level where there may be as little as 0.5 *μ*m between images in the 3D stack. The specific needs of the image data from our complex 3D cell culture model, being multichannel 3D image data where there are large distances between images in the 3D stack, require in-depth manipulation of the segmentation method that is not available within these platforms.

Markov random fields (MRFs) are used for computer vision in many different areas and for a broad range of problems [12]. Such models are popular because the Markov ‘neighbour’ structure allows for the spatial relations of the pixels to be taken into account while still being computationally feasible. For cellular microscopy image data, MRF based methodologies have been applied for tracking individual cells [13, 14], classification [15], motion detection [16], image restoration and deconvolution [17, 18], and identification of mitosis [19, 20]. MRFs have also been extensively used for image segmentation in a broad number of areas, with many ‘natural image’ (non-microscopy) applications [21]. For *in vivo* cellular microscopy images, MRFs have been used for segmentation in specific histology applications [22, 23] and for segmenting particular features such as blood vessels [24]. They have been used for *in vitro* cellular microscopy images to segment individual cell nuclei [25] including using a specific nuclei shape prior [26, 27]. An MRF based method has also been used for segmentation and classification of sub-cellular structures within individual cells [28].

We implement our 3D segmentation method based on MRFs and show that it performs accurately on image data from complex organotypic 3D models of prostate cancer. The strengths of our segmentation method are demonstrated by using different but closely related experimental image data and quantitatively comparing to other segmentation methods using ground truth labels. We show that our MRF based method accurately captures biologically relevant but often thinner and fainter features such as complex CAF structures and the distinction of tumour versus stroma cells in a 3D setting. We also show that our segmentation method is more generally applicable to other types of cellular microscopy image data and not only limited to fluorescence microscopy.

## Materials and methods

### Cellular microscopy image data

We utilise 3D stack confocal microscopy image data from three different but closely related organotypic 3D cell culture models, which we refer to as ‘IF image data’, the ‘LIVE image data’ and the ‘FAK image data’. Full details of the experimental and imaging protocols have been previously published [29] and are briefly outlined below. All 3D stacks of images from both the IF and LIVE image data were manually segmented by the biologists who performed the experiments. The manual segmentation was performed in Adobe Photoshop (Adobe Systems) and resulted in each pixel being given a ground truth label of either ‘in focus tumour cells’, ‘in focus CAFs’ or ‘out of focus/background’. These ground truth labels are used in the validation of our segmentation method.

Commercially available extracellular matrix preparations (growth factor-reduced Matrigel with a stock concentration of 8 mg/ml and collagen type-I with a stock 3 mg/ml) were purchased from BD Invitrogen and used for all 3D co-culture experiments. A 25% or 50% dilution of Matrigel was used and a 1:1 mixture of both preparations was routinely used (2–4 mg/ml Matrigel, 1.5 mg/ml collagen). Both tumour and stromal cells were seeded as single cell suspensions, with initial densities of 700–1500 cells/well. The 3D co-cultures were prepared using a ‘sandwich’ principle where all cells including stromal components were seeded in the same planar layer to facilitate imaging. Under these premises, initial seeding density was approximately 1800 cells/cm^2^.

In the IF 3D co-culture setting, LNCaP prostate cancer cells [30] were co-cultured with PF179T CAFs isolated from a prostate cancer patient [31], at an initial cell seeding ratio of 1:2 in extracellular matrix. After 14 days of culture, cells were fixed and indirect immunofluorescence staining was performed. An antibody specific for pan-keratin was used for the specific staining of tumour organoids, whereas CAFs were stained with an antibody against human vimentin. CAF cells eventually merged into large multicellular structures, which were characteristically surrounding the periphery of tumour organoids.

In the LIVE 3D co-culture setting, variations of the same cells as for the IF image data set were used. In this experiment, LNCaP tumour cells expressing DsRed protein were co-cultured with PF179T CAFs expressing GFP. The same experimental conditions, extracellular matrix extracts and other settings as above were used. The formation, growth, differentiation and morphogenesis of tumour organoids, as well as the formation of multicellular structures from single CAFs was monitored over a period of 14 days, after which imaging was performed. The FAK 3D co-culture setting was the same as the LIVE setting with the addition of focal adhesion kinase (FAK) inhibitors Y11 and PF-573228 purchased from Tocris Bioscience. The three conditions were: DMSO control (0.01%), 5 *μ*M concentration of Y11 and 5 *μ*M concentration of PF-573228.

Multichannel 3D confocal digital images were acquired where the tumour cells and CAFs were imaged separately. Both cell types are presented below in different (false) colour channels: red for tumour cells and green for CAFs. The IF and LIVE image data were acquired with a Zeiss Axiovert-200M microscope, equipped with a Yokogawa CSU22 spinning-disc confocal unit using a Zeiss Plan-Neofluar 20 × objective (numerical aperture 0.17). The IF image data consist of 8 stacks of images while the LIVE image data consist of 12 stacks of images. Each image has dimension 512 × 672 pixels and the number of images in a single stack ranges from 9 to 22. For all images from both data sets, one pixel ≈ 0.5 *μ*m within an image and the distance between adjacent images in a stack is 10 *μ*m. The FAK image data were acquired with the same microscope settings using a Zeiss Plan-Neofluar 5 × objective (numerical aperture 0.16) and consist of 24 stacks of images (8 for each condition). One pixel ≈ 2.5 *μ*m within an image and the distance between adjacent images in a stack is 40 *μ*m. Each image has dimension 512 × 672 pixels and the number of images in a single stack is 18.

An independent, lower complexity 2D image data set (BBBC003v1) provided in the Broad Bioimage Benchmark Collection [32] is also used for segmentation validation. The data consist of 15 images of single multicellular mouse embryos, each of which is a 640 × 480 pixel greyscale image captured with differential interference contrast microscopy and with a ground truth segmentation of the entire embryo structure. We additionally consider segmentation performance with the 2D phase contrast ‘Melanoma’ and ‘Tscratch’ image data (BBBC019), also from the Broad Bioimage Benchmark Collection. The Melanoma image data consist of 20 images each with dimension 1024 × 1280 pixels while the Tscratch image data consist of 24 images each with dimension 1028 × 1384 pixels. Both data sets are from ‘wound healing’ experiments with ground truth segmentations also provided.

### Segmentation method

**Fig 1:**
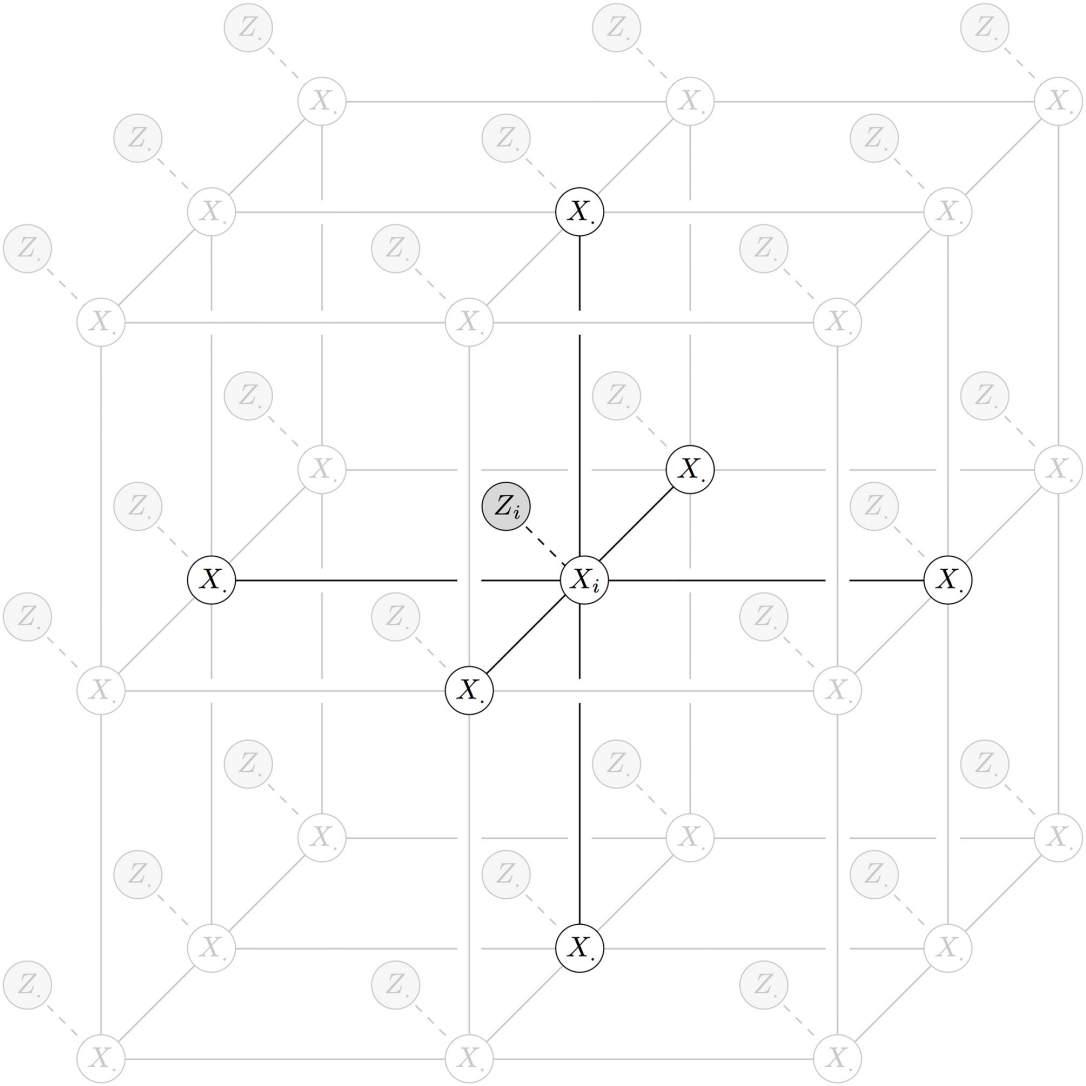
Conditional independence graph of a 6-neighbour conditional MRF with highlighted edges of the vertex corresponding to *X*_*i*_.

Within the Markov random field (MRF) framework, each pixel of a digital image is given a label from some predefined set (classically ‘foreground’ and ‘background’ for segmentation). We find the labelling that optimises a corresponding energy function so that the label of each pixel corresponds to the observed pixel value and so that there is a ‘smooth’ labelling across the whole image. For every pixel *i* in a digital image, let *X*_*i*_ and *Z*_*i*_ be the random variables for pixel label (unobserved) and pixel intensity (observed) respectively. Let the collection of random variables for all pixels have the conditional independence graph given in Fig 1. Then the collection of random variables is a conditional MRF with corresponding energy function

$$\begin{array}{c} E\left(x\right)=\sum_{i}\sum_{l}u_{i;l}I\left\{x_{i}=l\right\}+\sum_{\left(i,j\right)}\sum_{l}\sum_{k}w_{ij;lk}I\left\{x_{i}=l,x_{j}=k\right\} \end{array}$$ (1)

for pixels *i* and adjacent pairs of pixels (*i*, *j*) with labels *l* and *k*, and where *I* is the indicator function,

$$\begin{array}{c} I\left\{a=b\right\}=\left\{\begin{array}{cc} 1 & \text{if}\;a=b\\ 0 & \text{if}\;a\neq b. \end{array}\right. \end{array}$$ (2)

We find the minimum energy labels (segmentation)

$$\begin{array}{c} \hat{x}=\underset{x}{\mathrm{argmin}}\;E\left(x\right). \end{array}$$

The unary potentials *u*_*i*;*l*_ are defined to be

$$\begin{array}{c} u_{i;l}=-\log\left(\pi_{l}\left(z_{i}\right)\right) \end{array}$$ (3)

where *z*_*i*_ is the observed pixel value and π_*l*_ is the probability density function corresponding to label *l*. The pairwise potentials *w*_*ij*;*lk*_ are defined to be

$$\begin{array}{c} w_{ij;lk}=\left\{\begin{array}{cc} \;\;\frac{1}{\text{dist}(i,j)}\;\;\left(\lambda_{0}+\lambda_{1}\exp\{-\frac{1}{2\beta }\left|\right|z_{i}-z_{j}{\left|\right|}^{2}\}\right) & \text{if}\;l\neq k\\ 0 & \text{if}\;l=k \end{array}\right. \end{array}$$ (4)

where *z*_*i*_ and *z*_*j*_ are the observed pixel values, *λ*_0_, *λ*_1_ and *β* are parameters to be set, and dist(*i*, *j*) is the distance between pixels *i* and *j*. Note that the distance may be different for neighbouring pixels within an image compared to neighbouring pixels between images depending on the resolution of the image data in each dimension.

The vertices and edges of a conditional independence graph encode the conditional independence properties of the corresponding set of random variables. In the conditional MRF graph (Fig 1), every edge corresponds to either a unary (*X*_*i*_, *Z*_*i*_) or pairwise (*X*_*i*_, *X*_⋅_) potential in the corresponding energy function Eq (1). Fig 1 shows a 6-neighbour conditional MRF (6 pairwise potentials for each pixel), which we use for our 3D segmentation method. Each pixel has 4 neighbours within an image and 2 neighbours between images in the 3D stack, with less neighbours for pixels on the borders of the stack.

The unary and pairwise potentials establish the correspondence and ‘smoothness’ of the overall pixel labelling respectively. The parameters *λ*_0_, *λ*_1_ and *β* determine the amount of influence that the pairwise potentials have over the unary potentials in the minimisation of the energy function (1). The trade-off is that we require a labelling with ‘smooth’ contiguous segmented regions (neighbouring pixels have the same label) but also want to preserve discontinuities present in the image (S1 Fig). The form of the unary and pairwise potentials is standard for the MRF segmentation problem [33]. The potentials satisfy the sub-modularity condition which guarantees the existence of a minimum energy solution in the binary label case and allows for an approximation of the minimum energy solution in the multi-label case [34].

**Fig 2:**
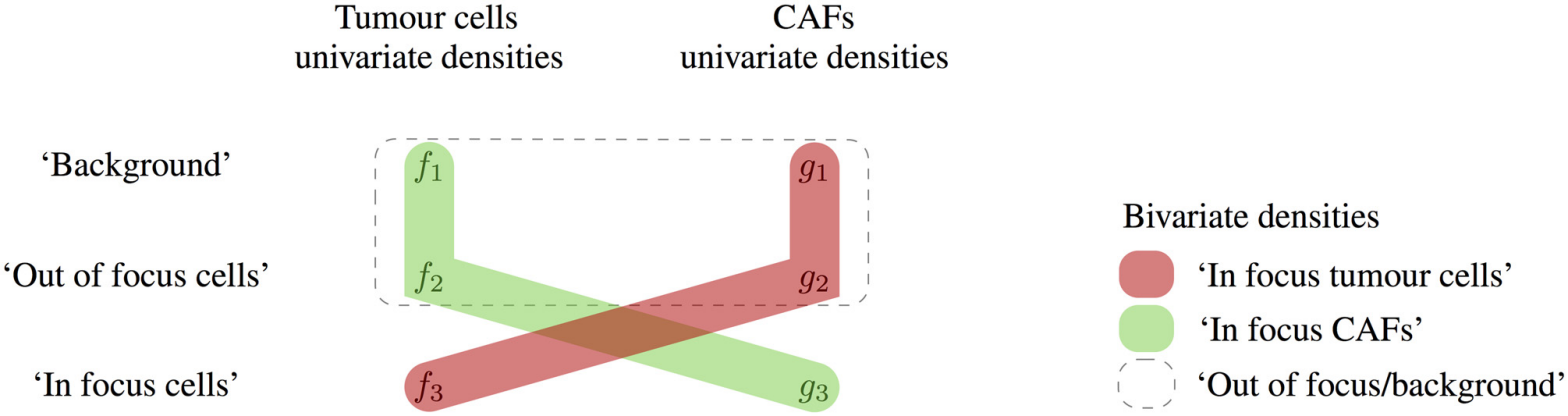
Schematic diagram of the construction of the densities for each label. The three labels are: ‘In focus’ tumour cells with a mixture of ‘out of focus’ CAFs and ‘background’ in the other dimension (red), ‘in focus’ CAFs with a mixture of ‘out of focus’ tumour cells and ‘background’ in the other dimension (green) and a mixture of ‘out of focus’ and ‘background’ in both dimensions (dashed).

In order to set the unary potentials Eq (3) we require a density function π_*l*_ over the pixel values corresponding to each label *l*. The standard method is to employ an ‘interactive segmentation’ procedure and have the user manually ‘seed’ regions of the image for each label [33]. Then, the empirical distributions of the seeded regions are used to calculate the unary potentials. Instead of manually seeding the image, we fit a univariate Gaussian mixture model with three components to the density of the observed pixel values in the red and green channels separately. The densities used for the segmentation labels are then bivariate mixtures of the univariate components obtained in each colour channel (Fig 2).

The parameters *λ*_0_, *λ*_1_ and *β* are set based on the distributions of the unary potentials to balance the trade-off between correspondence and smoothness in the segmentation output. Although there are methods for learning these parameters using a training set of ground truth labelled image data, these methods are highly computationally expensive and so not often utilised in practice [35]. Moreover, a ground truth labelling is rarely available in most applications. For each stack of images we set

$$\begin{array}{c} \lambda_{0}=\min_{i,l}u_{i;l} \end{array}$$

and

$$\begin{array}{c} \lambda_{1}=\max_{i,l}u_{i;l}-\lambda_{0} \end{array}$$

so that the unary and pairwise potentials share the same range. The value of *β* is set manually on a candidate stack of images and the same value is used for all other stacks from the same experiment.

As a pre-processing step, we use a local entropy filter [9] to quantify local texture (built-in function entropyfilt in the MATLAB Image Processing Toolbox). The local entropy filter is applied to both the red and green channels separately. The raw greyscale images are down-sampled to 8-bit so that each pixel directly corresponds to an intensity value in the set {0, 1, …, 255}. For each pixel *i*, the local entropy filtered value is

$$\begin{array}{c} \eta_{i}=-\sum_{j\in N_{i}}p_{j}\log_{2}\left(p_{j}\right) \end{array}$$

where *p*_*j*_ is the proportion of pixels in the neighbourhood $N_{i}$ that have equal intensity to pixel *j*. The neighbourhood $N_{i}$ of each pixel *i* is the 9 × 9 square centred on pixel *i* inclusive with symmetric padding around the border of the image.

**Fig 3:**
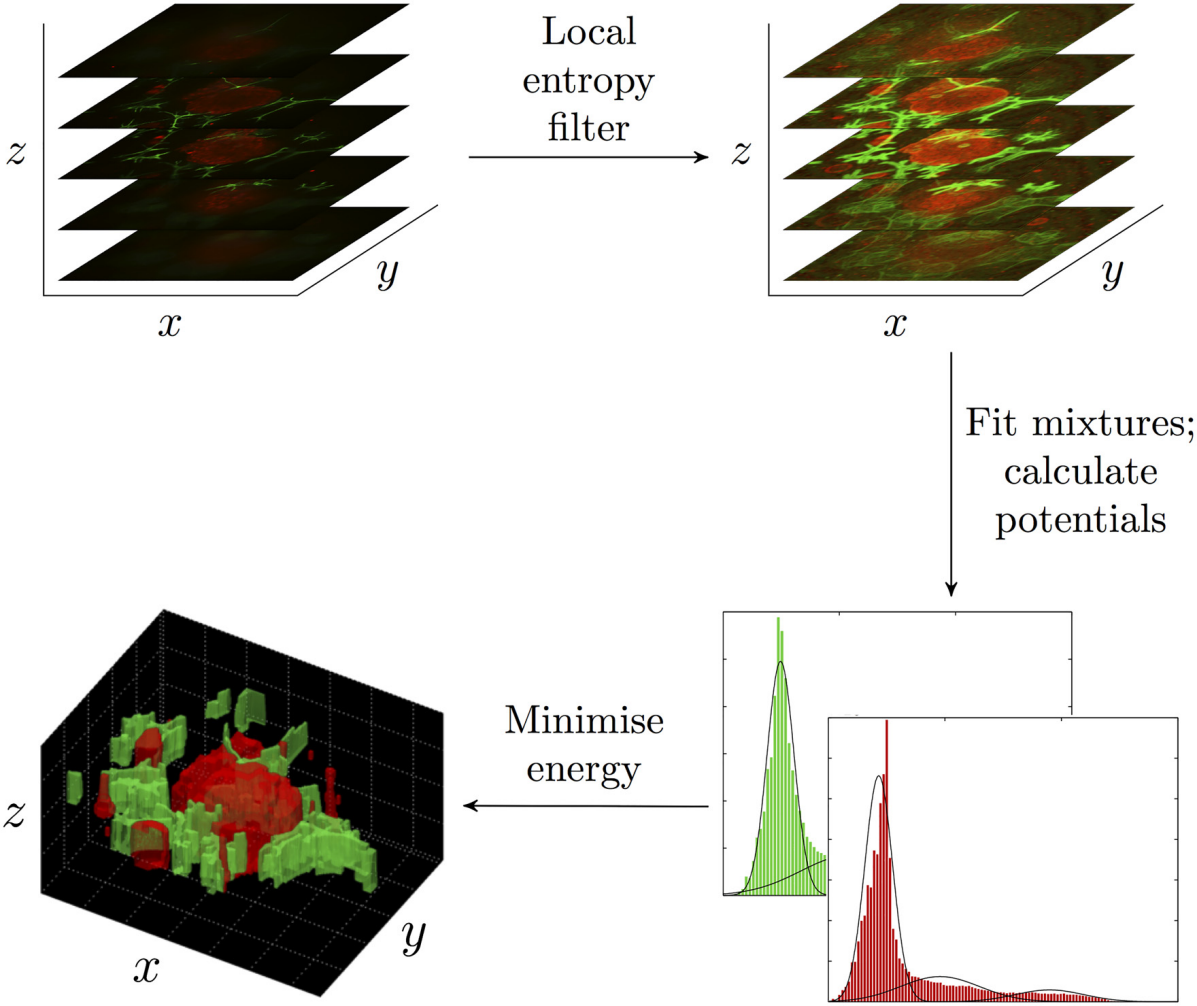
Overview of our 3D segmentation method. Confocal microscopy is used to image 3D co-culture models resulting in a 3D stack of images. Our 3D segmentation method is applied to the 3D stack of images resulting in 3D segmentation output.

Our 3D segmentation method is summarised in the following steps (Fig 3):

1. Process the 3D stack of images using the local entropy filter in the red (tumour cells) and green (CAFs) channels separately,
2. Fit univariate Gaussian mixture models to the filtered pixel values in the red (tumour cells) and green (CAFs) channels separately,
3. Combine the mixture densities (Fig 2) and calculate the unary and pairwise potentials,
4. Find the minimum energy labelling.

We implemented our segmentation method in MATLAB and used the *α*-expansion algorithm to find the minimum energy labelling [34, 36, 37].

### Validation method

No single tool is available to perform the required segmentation for any given data set. Each method has its own advantages and disadvantages and is often tailored to specific applications such as using prior shape information when segmenting single cells or cell nuclei [25–27]. Although many sophisticated segmentation methods exist, we are not aware of any that would be suitable to apply to the 3D stack image data from our complex 3D cell culture model. Hence for purposes of comparison, we use a series of standard segmentation methods that can be considered to ‘build up’ to our MRF approach:

1. **intOtsu** Using Otsu’s method [38] to threshold the red and green intensity channels separately and combining the labels so that ‘tumour cell’ or ‘CAF’ overwrites ‘background’, or if a pixel is labeled both ‘tumour cell’ and ‘CAF’ it is randomly assigned one of those labels.
2. **entOtsu** Using Otsu’s method to threshold the local entropy filtered red and green channels separately and combining the labels using the same procedure as above.
3. **mixtures** Thresholding the local entropy filtered images using the bivariate mixture densities (Fig 2).
4. **MRF** The method presented in this paper.

Otsu’s method finds the global threshold that maximises the inter-class variance of the resultant groups and is a widely used technique for basic greyscale image segmentation [9]. Mixture models are themselves also widely used for many image segmentation applications [39]. Since the bivariate mixtures are used to calculate the unary potentials in our MRF based segmentation method, the ‘mixtures’ method is equivalent to the MRF method presented in this paper with *λ*_0_ = *λ*_1_ = 0.

The output of the four segmentation methods is compared to the ground truth labels using the overall macro F_1_-score, the harmonic mean of the classification precision and recall [40]. Hence F_1_-score = 1 for a perfect labelling. The IF and LIVE image data as well as the independent mouse embryo image data are used for validation.

## Results and Discussion

We present a series of results in order to discuss the benefits of our MRF based method for our particularly complex biological application, in particular, multichannel 3D image data with large distances between images in the stack. Using the 3D segmentation output, it is possible to investigate if tumour cells and CAFs are forming cell-cell contacts or are physically separated in our 3D co-culture model, which has important implication for tumour biology. We additionally show the utility of the local entropy filter and that our MRF based approach obtains the most accurate segmentation results. The code for our segmentation method, the image data and the ground truth labels are all provided as supplementary material (S1, S2 and S3 Files).

### Segmentation output suggests the physical contact of multicellular tumour and CAF structures

The output of our 3D segmentation method can be used to investigate if multicellular tumour organoids and CAF structures are in direct contact.

Using confocal microscopy to image 3D cell culture results in a 3D stack of images corresponding to a series of parallel focal planes. A maximum intensity projection can be obtained by projecting the maximum intensity pixel values into the (*x*, *y*) plane. Although a substantial amount of information may be lost, the prospective 2D image analysis is much more developed and less computationally expensive than considering the entire 3D stack of images. For these reasons, current image analysis frameworks for 3D cell culture models of prostate cancer are based on maximum intensity projections [41, 42]. Although maximum intensity projections may be suitable to analyse monoculture models for certain applications, they are not appropriate for more complex organotypic models such as co-culture models. Since all structural information is projected into the (*x*, *y*) plane, it would not be possible to determine if tumour organoids and CAFs are in direct contact or physically separated using a 2D analysis.

**Fig 4:**
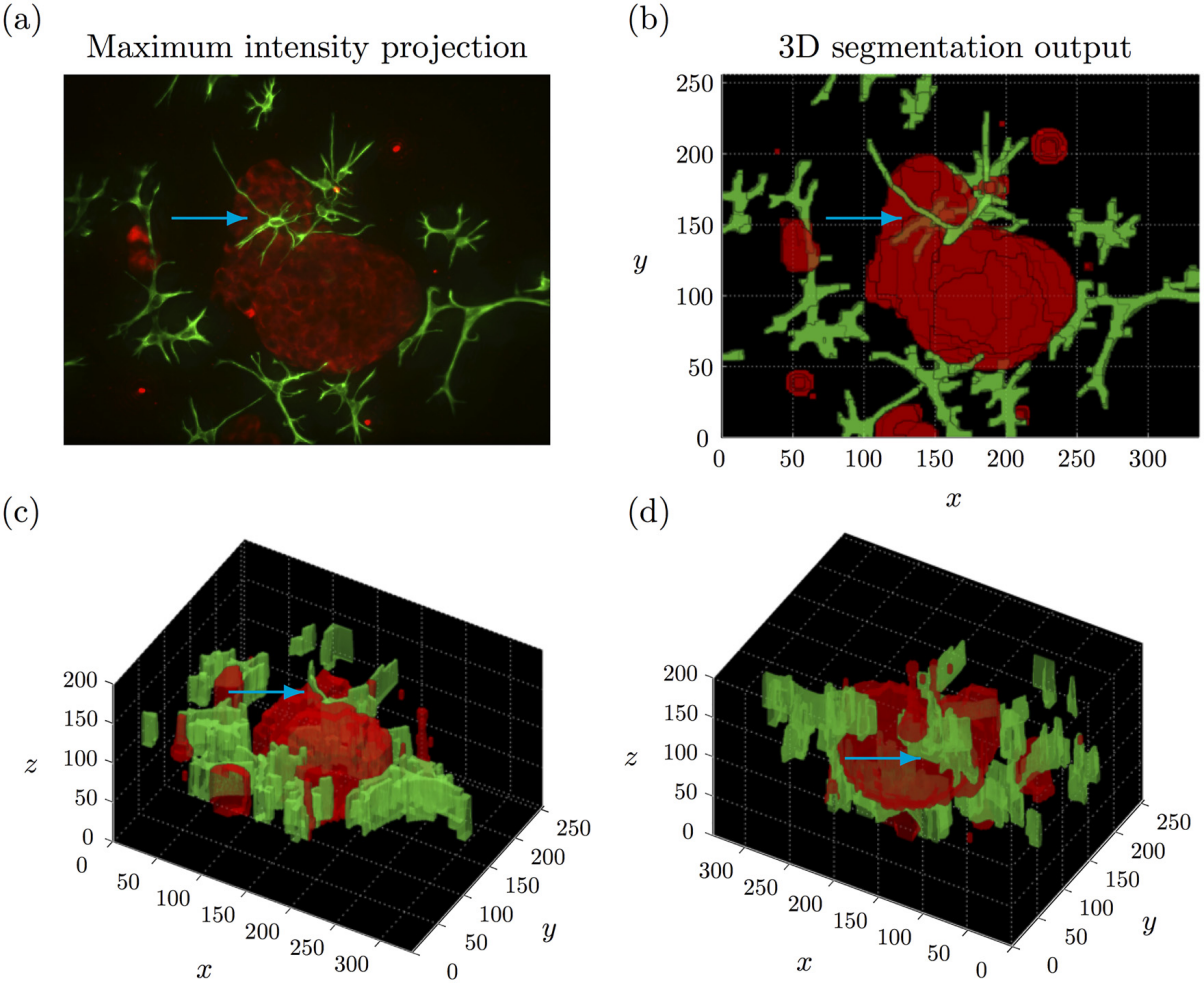
Example 3D segmentation output. (a) Maximum intensity projection of a representative 3D stack. (b)-(d) Different viewpoints on the corresponding 3D segmentation output using our MRF based method. The blue arrow indicates where the CAFs appear to be inside the tumour organoid in the maximum intensity projection but can be seen to be outside in the 3D segmentation output. See S1 Video for a video of the 3D segmentation output.

Fig 4(a) shows the maximum intensity projection of both the red and green channels (false colour) for an example 3D stack. It remains unclear if the central tumour and CAF structures are directly in contact. The CAF structures appear to be located within the tumour structure (blue arrow), which is biologically irrelevant. In clinical tumour samples, fibroblasts are not typically found within tumour organoids, but characteristically only surround epithelial (tumour) aggregates. Only occasionally, CAFs may come into contact with tumour cells at the surface of tumour structures.

The 3D segmentation of the tumour cells (red) and CAFs (green) is illustrated in Fig 4(b) and 4(d). The segmentation output specifically highlights those segmented regions corresponding to CAF structures that are in direct contact with the segmented regions corresponding to multicellular tumour structure. In particular, the CAFs that appeared to be located inside the tumour structure in the maximum intensity projection (Fig 4(a)) can now be seen to be outside, but in contact with either side of the central tumour organoid (blue arrow). S1 Video provides additional viewpoints on the 3D segmentation.

The 3D rendered segmentation output (Fig 4(b) and 4(d)) looks rather ‘blocky’, especially in *z* where the tumour organoid has a ‘flat top’. This is a feature of the image data and is due to the relative sparsity of the data in *z*. It would not be appropriate to extrapolate the rendering so that the tumour organoids look more like ‘round spheroids’ in 3D as this would potentially confound important structural information and would not be supported by the resolution of the image data.

It is possible to manually identify if and which segmented CAF regions are in direct contact with the segmented tumour organoids with the visualisation provided of our 3D segmentation output. In order to demonstrate a proof of concept of automatically quantifying tumour-stroma contact with the segmentation output, we consider the FAK image data (S2 Fig). With our MRF segmentation, we consider that the contact between the ‘tumour cells’ and ‘CAFs’ segmented regions will give an estimate of the tumour-stroma contact in the model.

**Fig 5:**
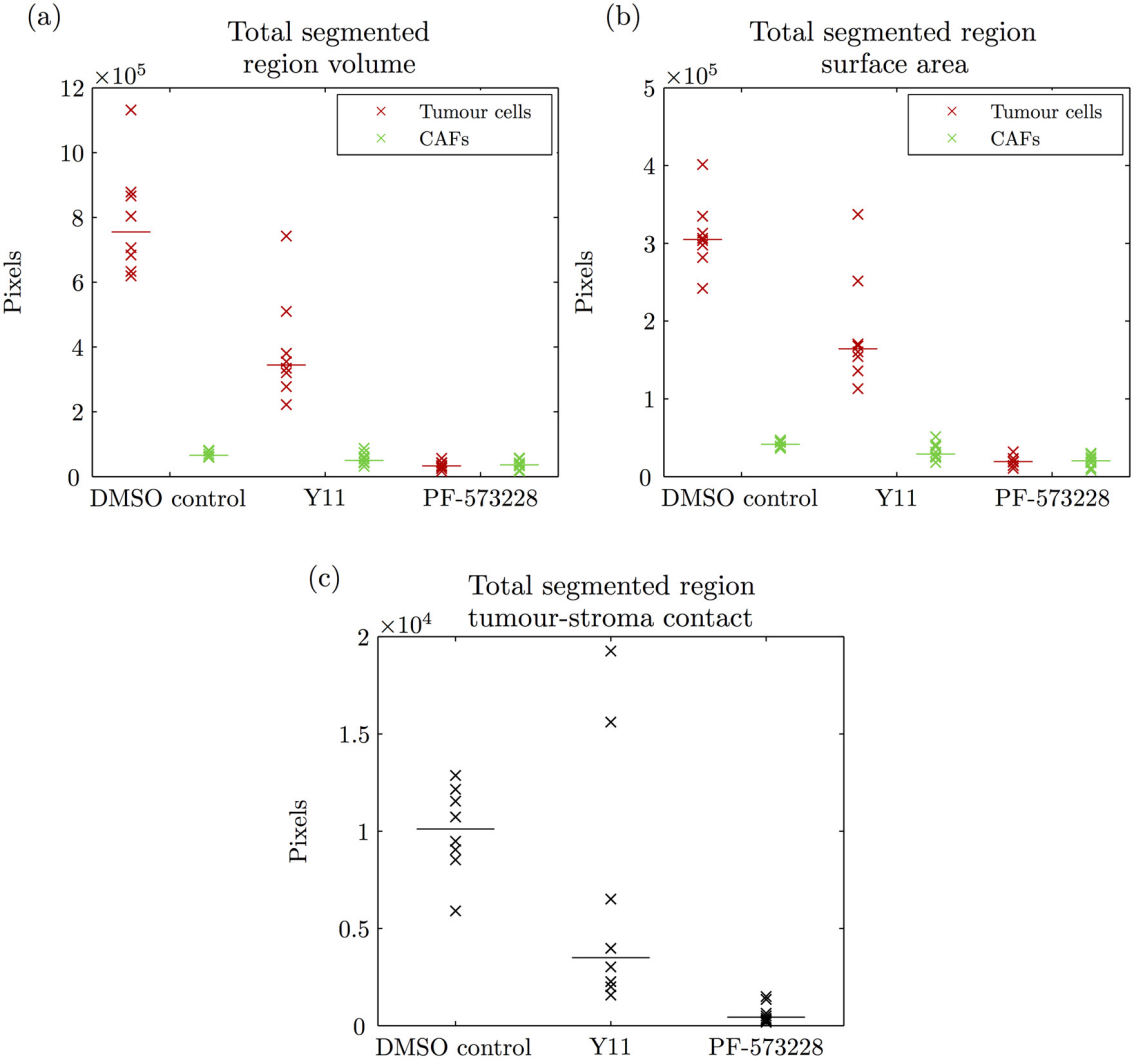
Quantification of tumour-stroma contact in the FAK image data. (a)-(b) Scatter plots of the total volume and total surface area (in pixels) of the segmented ‘tumour cells’ (red) and ‘CAFs’ (green) regions. (c) Scatter plot of the total contact (in pixels) between the segmented ‘tumour cells’ and ‘CAFs’ regions.

Fig 5 shows scatter plots of total volume, total surface area and total contact for the segmented ‘tumour cells’ and ‘CAFs’ regions in the FAK image data. Total volume is the total number of labelled pixels, total surface area is the total number of labelled pixels adjacent to a pixel with another label and total contact is the total number of labelled ‘tumour cells’ and ‘CAFs’ pixels that are adjacent. Total volume and total surface area of both the tumour organoids and CAF structures are generally smaller upon FAK inhibition (Fig 5 (a) and 5(b)) and this conforms to previous studies [29]. DMSO control displays a baseline of tumour-stroma contact (Fig 5(c)) and the FAK inhibitors clearly disrupt this contact. With the inhibitor Y11, there appears to be less contact on average, however the contact is also more variable. There are two image stacks that have higher total volume, surface area and contact in particular (indicated by blue asterisks in S2 Fig). It is clear that PF-573228 inhibits tumour organoid growth (Fig 5(a) and 5(b)) and hence there are few physical contacts between the two cell types (Fig 5(c)). A Kruskal-Wallis test was performed on total contact (Fig 5(c)) and the null hypothesis that the different samples are all realisations of the same distribution was rejected (p-value = 2.6 × 10^−4^). Corresponding pairwise comparisons were performed using the Mann-Whitney *U* test. A significant difference in total contact between PF-573228 and the others was found (p-values <1.6 × 10^−4^) while there was no significant difference found between DMSO control and Y11 (p-value = 0.13).

It is possible that the individual tumour organoids and CAF structures could be evaluated individually rather than considering the total quantities for each 3D stack. This opens up the possibility for more detailed statistical shape analysis to further investigate tumour-stroma contact, which is the subject of future work. However, it is also possible that our segmentation method could be used in different applications with multichannel image data where there are multiple cell types of interest and experimental aims other than investigating cell contact.

In principle, both red and green colour channels could be processed, segmented and analysed separately using our approach. The demonstration of our segmentation method on greyscale images is presented below using the mouse embryo image data. In this case, since tumour and stromal counterparts are likely to influence each other within the 3D model, both channels were analysed together. In particular, this is necessary to investigate whether cell-cell contacts between tumour and stromal cells occur at all and if these contacts are critical for forming the corresponding tissue-like structures *in vitro*. From a mathematical point of view, our method could be used to segment cellular microscopy image data with *n* false colour channels by performing the preprocessing steps on each colour channel separately and suitably defining the multivariate mixtures.

It is conceivable that in future studies on organotypic 3D cell culture models, such automatic segmentation output may provide valuable information related to the response of tumour tissues, including the tumour microenvironment and the stromal component, to therapeutic agents. As we have demonstrated that cell-cell contact between both cell types apparently occur and can be automatically quantified, our approach is suitable for applications in which the nature of tumour-stroma cell-cell interactions contact can be functionally tested. For example, such contact is likely to be critical for the response of tumour cells to anti-cancer chemotherapeutic drugs.

### Utility of the local entropy filter

We show that the issue of inhomogeneous fluorescence of both the tumour organoids and CAF structures, as well as the potential inhomogeneous light illumination in both channels, is addressed by using the local entropy filter.

In our protocol, all multicellular organoids are considered of equal importance as each potentially carries morphological information that reflects the genetic variability contained in a certain tumour cell line. In the context of cell and tissue based models for cancer biology, multicellular tumour structures often display significant heterogeneity. Cellular heterogeneity is expected to be of critical relevance in clinical cancers, where this aspect represents a key risk factor for cancer progression and therapy failure. To capture this critical heterogeneity we require a segmentation method that assigns a ‘tumour cells’ label to each tumour organoid regardless of their different fluorescence intensities and multicellular morphologies (size and shape). This is equally true for when the multicellular structures appear to have different fluorescence intensity due to inhomogeneous light illumination in the microscope.

CAFs also exhibit heterogeneous fluorescence as well as forming complex multicellular structures which are more challenging to segment as a whole. The complex shapes of the CAF structures are fundamentally different from tumour organoids, which are typically round spheroids. However it is equally biologically relevant to segment the CAF structures in their entirety. Additionally, such complex shapes need to be segmented simultaneously with the round tumour spheroids so that the segmentation output can in principle be used to quantify the physical contact of tumour and CAF structures.

**Fig 6:**
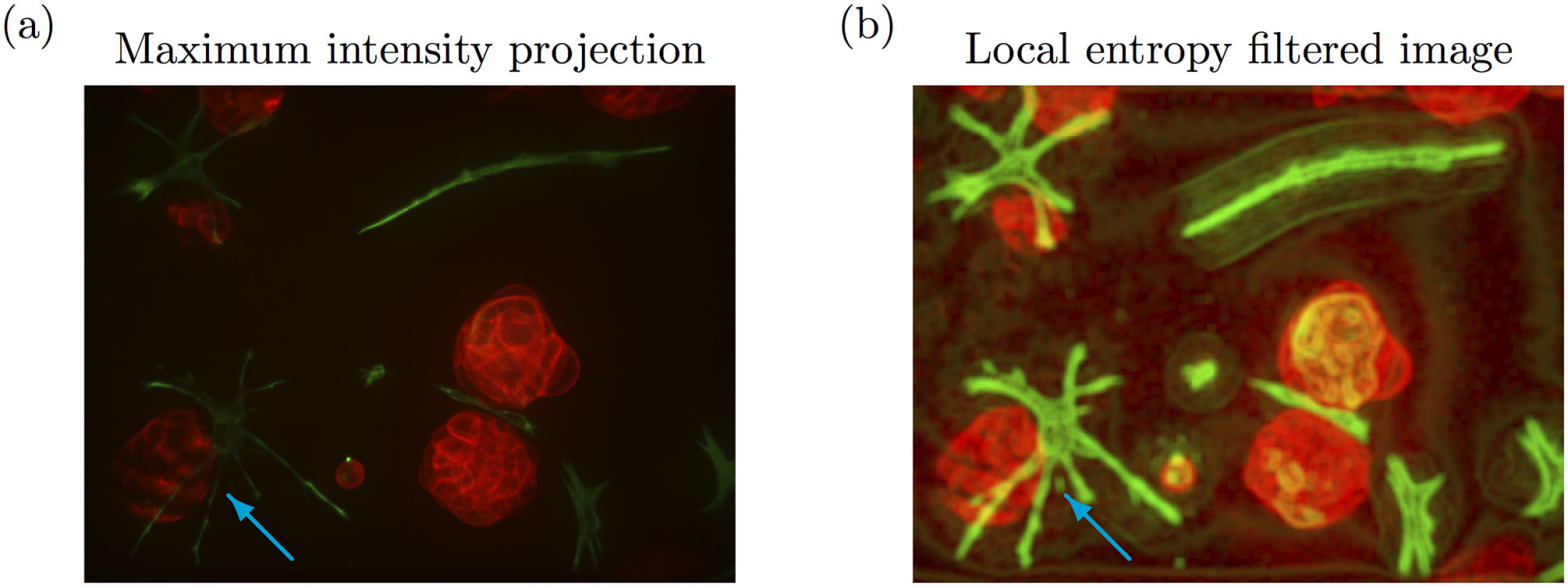
Utility of the local entropy filter. (a) Maximum intensity projection of an example 3D stack. (b) The corresponding local entropy filtered image. The blue arrow indicates where a weakly fluorescent tumour organoid and CAF structure is more clearly distinguished in the local entropy filtered image.

The above issues are illustrated in Fig 6. The multicellular tumour structure on the bottom left of Fig 6(a) (blue arrow) appears to have different fluorescence intensity than the two more central structures. Additionally, the CAF structure directly adjacent to this tumour organoid appears to be more weakly fluorescent compared to the other CAF structures in the same image. The local entropy filtered image of both the red and green channels of the maximum intensity projection is shown in Fig 6(b). The local entropy filter clearly distinguishes the texture that is shared by all the multicellular tumour structures, regardless of their variable fluorescence intensity. Additionally, it appears that the connectivity of the CAF structures is much more readily apparent than in the original maximum intensity projection (blue arrow).

**Fig 7:**
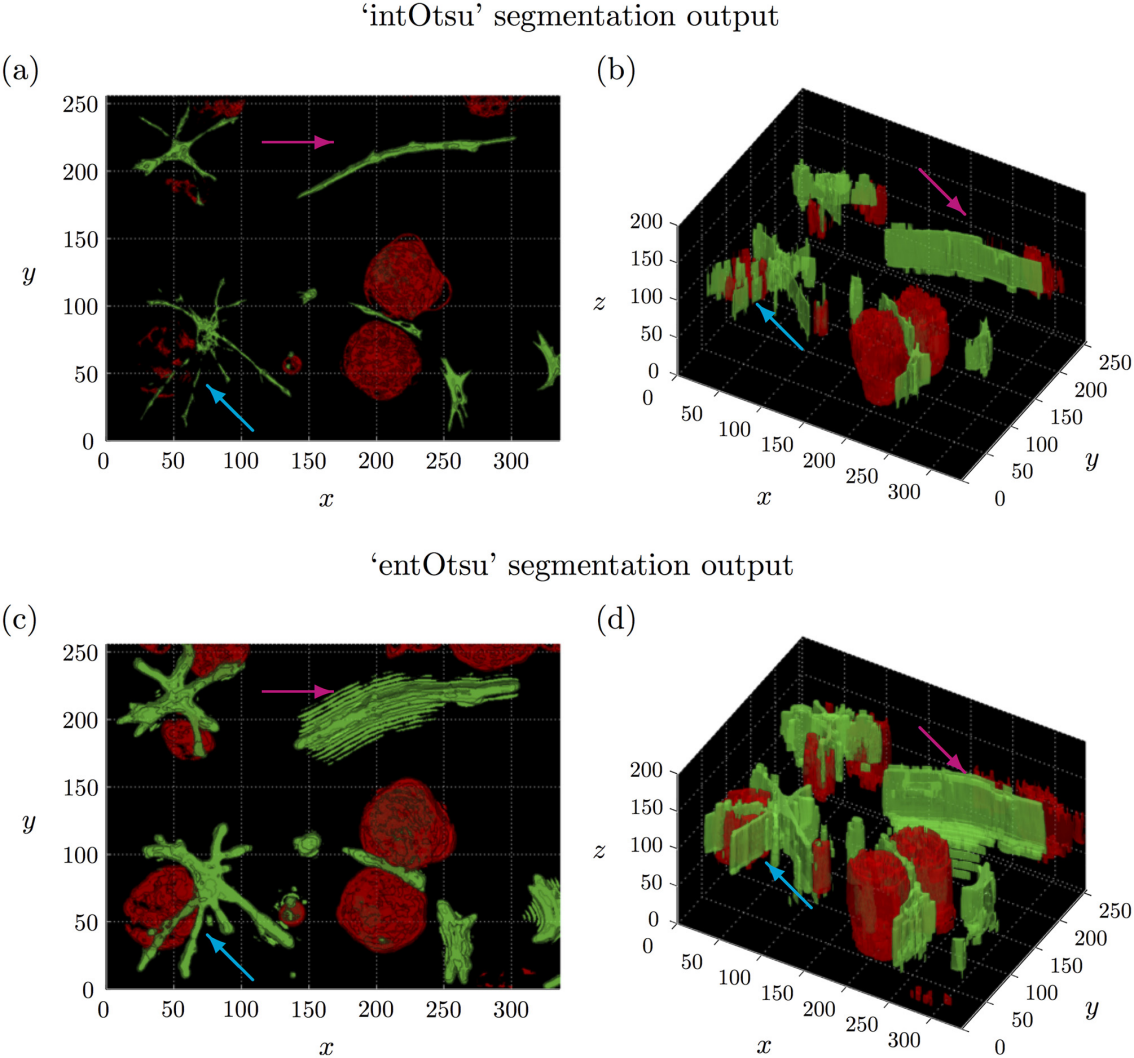
Comparison of 3D segmentation output with and without the local entropy filter. (a)-(b) 3D segmentation output using the ‘intOtsu’ method on the original intensity images. (c)-(d) 3D segmentation output using the ‘entOtsu’ method, which includes using the local entropy filter. The blue arrow indicates where a tumour organoid and CAF structure appears to be segmented more accurately by the ‘entOtsu’ method. The magenta arrow indicates where the ‘entOtsu’ method appears to have segmented a different CAF structure less accurately.

Fig 7(a) and 7(b) shows the 3D segmentation output on the corresponding 3D stack using the ‘intOtsu’ method while Fig 7(c) and 7(d) shows the 3D segmentation output using the ‘entOtsu’ method, which includes using the local entropy filter. Otsu’s method chooses a global threshold and thus only the brightest parts of the tumour cell and CAF structures are labelled. In the ‘entOtsu’ segmentation output, all of the multicellular tumour structures are identified along with the CAF structures, which is not the case in the output of the ‘intOtsu’ segmentation (blue arrow). However in the ‘entOtsu’ segmentation output, some CAF structures appear less accurately segmented (magenta arrow). Note that the maximum intensity projection and corresponding local entropy filtered images (Fig 6) were not used as input for either segmentation method. These visualisations of the 3D stack are presented for comparison to the 3D segmentation output.

### Validation results

**Fig 8:**
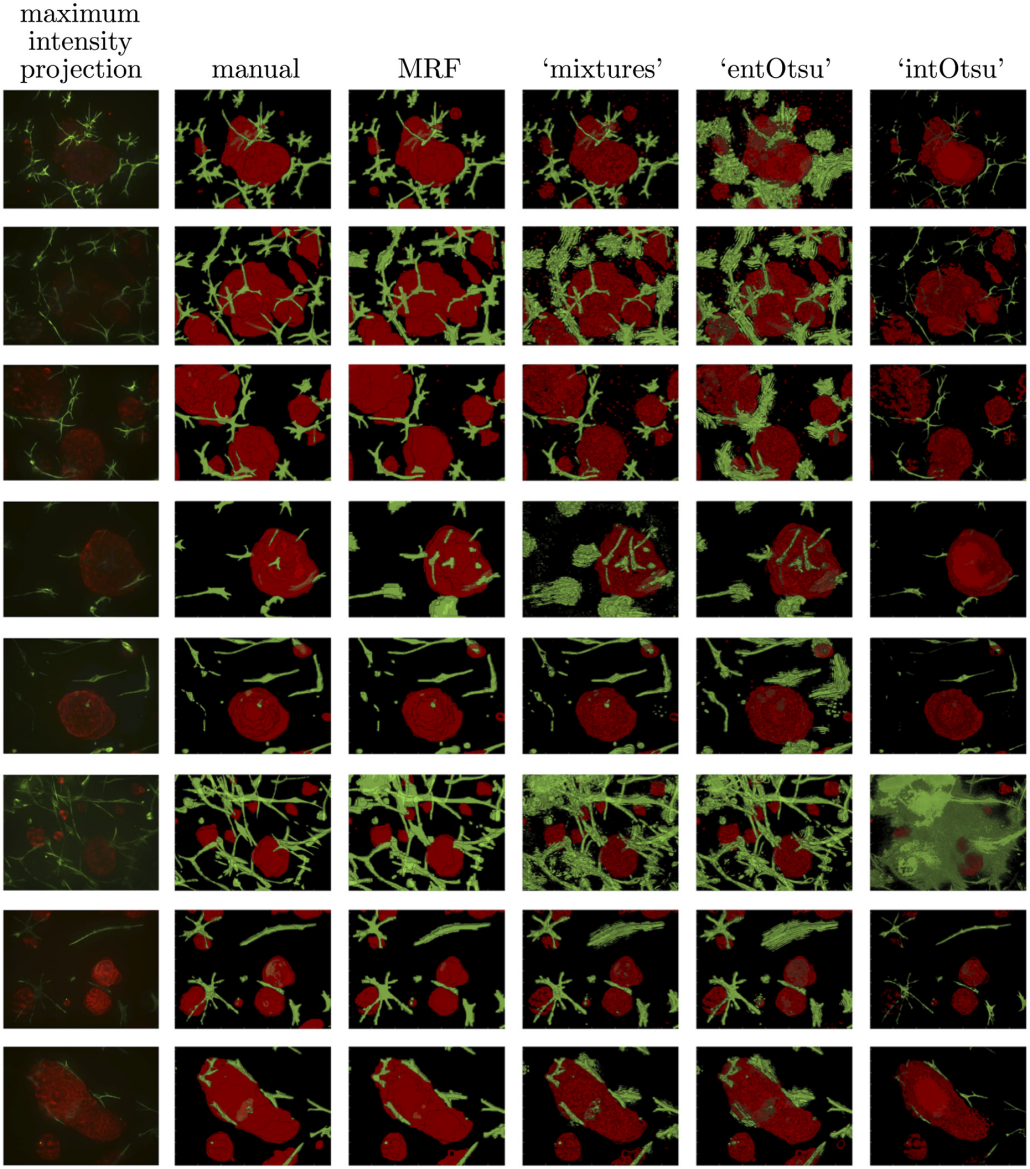
Maximum intensity projections, manual segmentations and the output for all segmentation methods on the IF image data.

**Fig 9:**
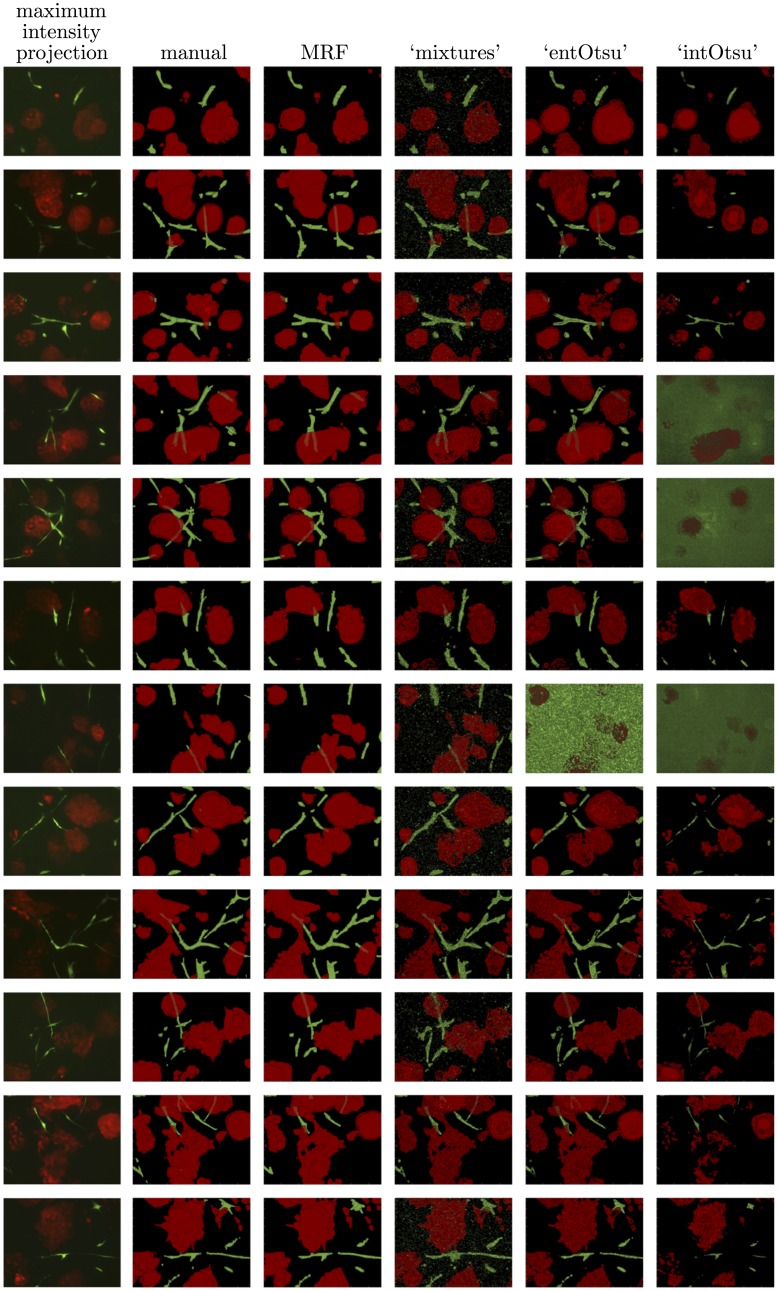
Maximum intensity projections, manual segmentations and the output for all segmentation methods on the LIVE image data.

**Fig 10:**
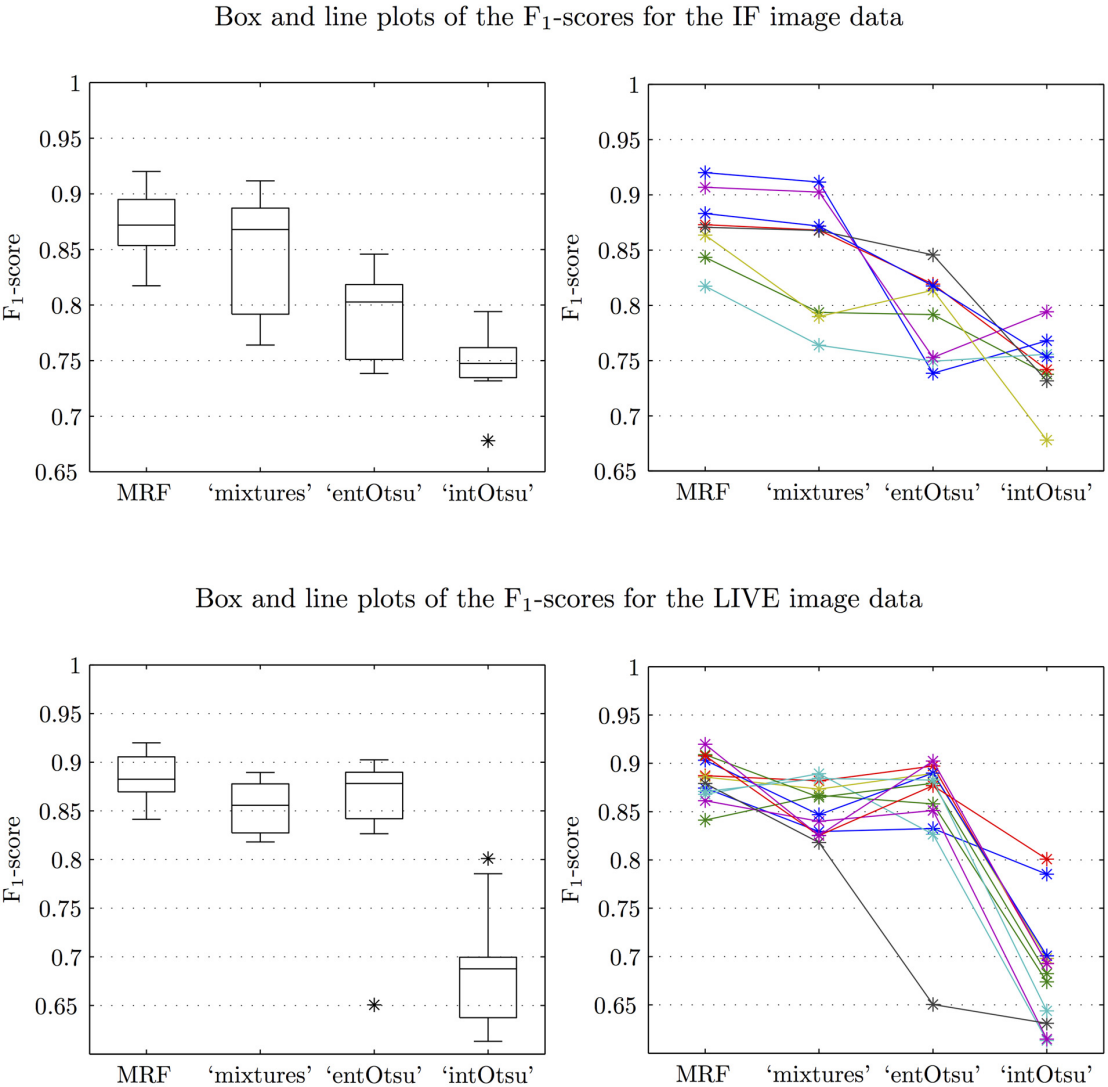
Box and line plots of the F_1_-scores for the IF and LIVE image data.

**Fig 11:**
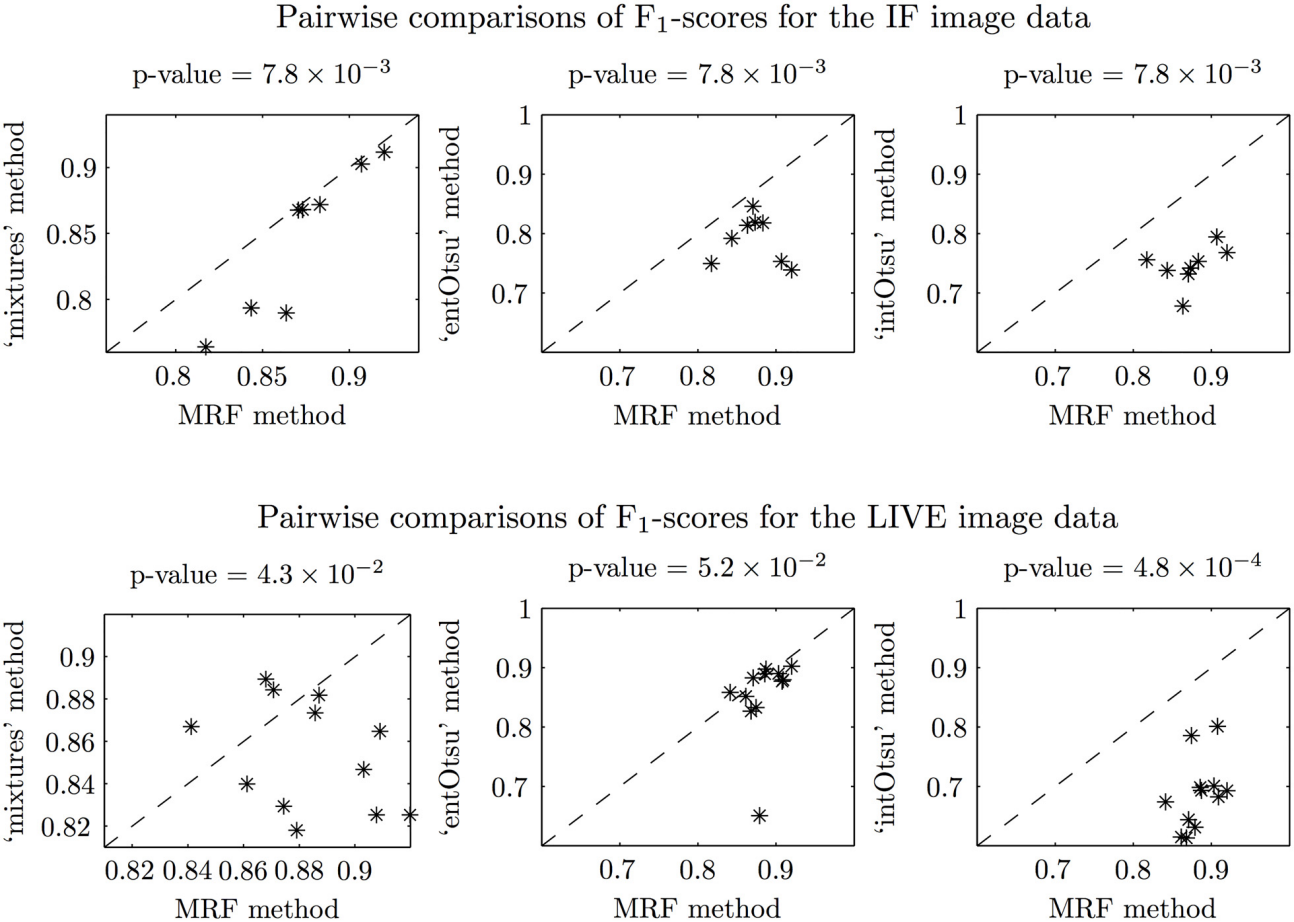
Pairwise comparisons of the F_1_-scores for the IF and LIVE image data. The dotted lines correspond to equal F_1_-score and the p-values correspond to Wilcoxon signed-rank tests that there is no difference between the paired F_1_-scores. Note that for the IF image data, the p-values are all equal because the MRF F_1_-score is always higher (all points are below the dotted line). For the comparison with the ‘intOstu’ method for the LIVE image data, the MRF F_1_-score is also always higher but the p-value is lower here because there are more data points.

Segmentation output for the IF and LIVE image data is given in Figs 8 and 9 respectively. The mean (and median) macro F_1_-score for each segmentation method is shown in Table 1. These scores generally follow the way that the methods ‘built up’ to our MRF based approach. Box plots of the F_1_-scores are given in Fig 10 and since the data are not independent across the different segmentation methods, corresponding line plots are also given. Pairwise comparisons are shown in Fig 11 and the reported p-values correspond to a Wilcoxon signed-rank test with the null hypothesis that there is no difference between the paired F_1_-scores. Our MRF based method generally has significantly higher F_1_-score compared to each of the other methods (Fig 11). This is clearly shown for the IF image data, but it is not as clear for the LIVE image data. A possible explanation for this difference in performance is that the CAF structures in the LIVE image data are noticeably less complex than in the IF image data (Figs 8 and 9). On the less complex image data the MRF, ‘mixtures’ and ‘entOtsu’ methods all give closer results.

**Table 1:** Mean (and median) macro F_1_-scores of the segmentation methods for the IF and LIVE image data.

	MRF	‘mixtures’	‘entOtsu’	‘intOtsu’
IF image data	0.87 (0.87)	0.85 (0.87)	0.79 (0.80)	0.75 (0.75)
LIVE image data	0.88 (0.88)	0.85 (0.86)	0.85 (0.88)	0.69 (0.69)

We have qualitatively shown the utility of the local entropy filter for quantifying the texture of both the multicellular tumour and CAF structures. The main difference in performance between the ‘entOtsu’ and ‘mixtures’ methods is that with the ‘entOtsu’ method, a threshold was taken on each colour channel separately and the two binary segmentation outputs were combined in a necessarily ad-hoc manner. The construction of the bivariate densities gives a principled way to combine the multiple colour channels and achieve a multi-label segmentation.

**Fig 12:**
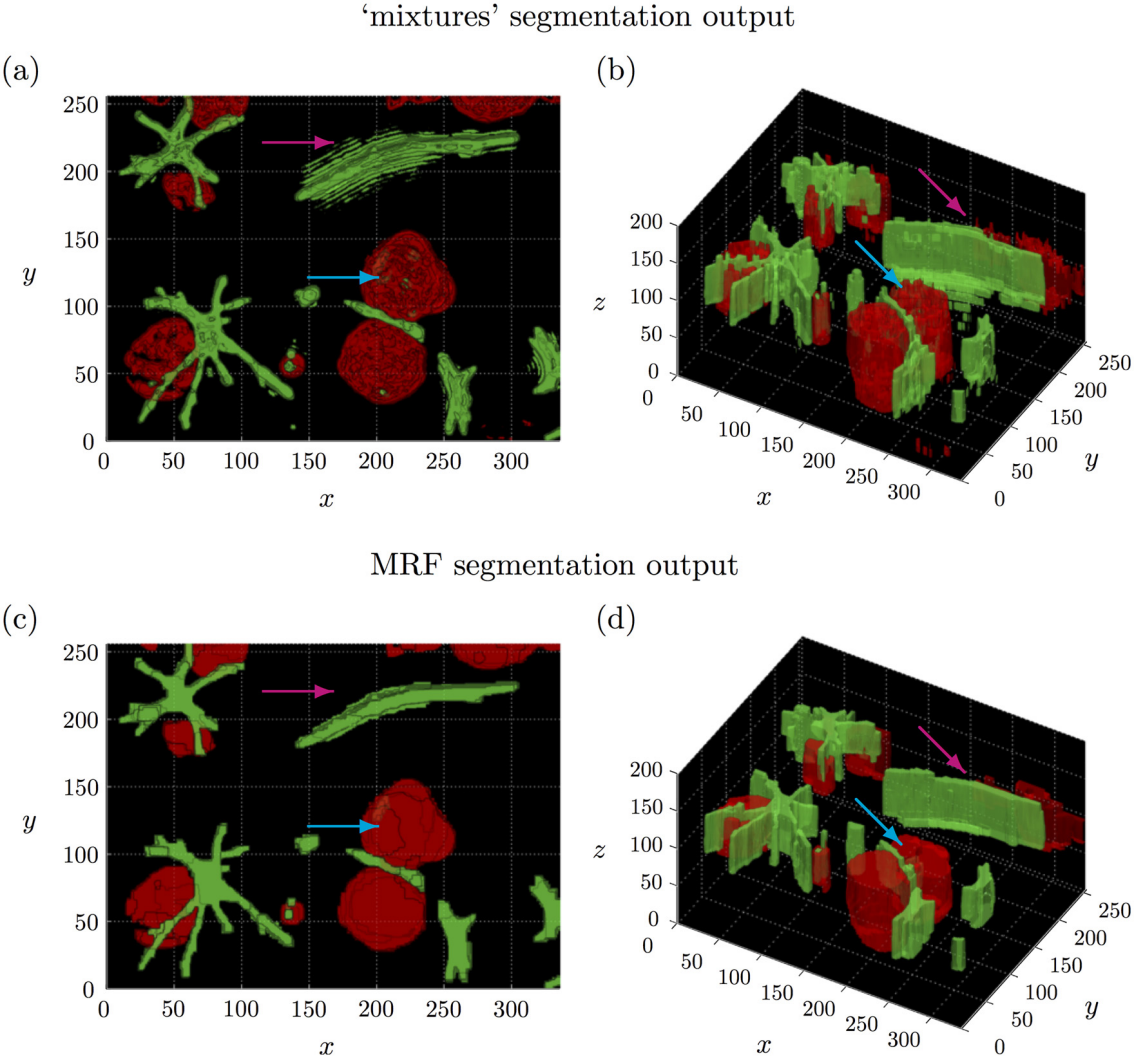
Comparison of 3D segmentation output with and without the ‘neighbour’ information. (a)-(b) 3D segmentation output using the ‘mixtures’ method, which is equivalent to our MRF based method with *λ*_0_ = *λ*_1_ = 0. (c)-(d) 3D segmentation output using our MRF based method with *λ*_0_ = min_*i*, *l*_ *u*_*i*;*l*_, *λ*_1_ = max_*i*, *l*_ *u*_*i*;*l*_ − *λ*_0_ and $\beta =\frac{1}{40}$. The blue and magenta arrows indicate areas where the MRF method produces a smoother segmentation. See S2 Video for a video of the 3D segmentation output.

Following the discussion above, we can see that the ‘intOtsu’ method tends to under-segment the multicellular structures whereas the ‘entOtsu’ and ‘mixtures’ methods generally appear to over-segment the multicellular structures (Figs 8 and 9). In particular the ‘roughness’ of these segmentation methods is readily apparent and many individual pixels are assigned a different label to their neighbours. This is particularly clear in the *z* dimension, where the front face of the segmented tumour organoid regions appear especially rough. This is apparent in Fig 12 (blue arrow), as is that the rough ‘mixtures’ segmentation of the top centre CAF structure is also accounted for with the MRF (magenta arrow). Note that the corresponding maximum intensity projection of the image data in Fig 12 can be seen in Fig 6. The MRF method provides a principled way to achieve a smooth segmentation of multiple objects of interest without assuming any prior shape information on either the multicellular tumour organoids nor the multicellular CAF structures. Note that these details are not readily captured by the F_1_-score, which was 0.871 and 0.868 for the MRF and ‘mixtures’ segmentations given in Fig 12 respectively.

To further validate our MRF based segmentation method, we considered the independent mouse embryo, Melanoma and Tscratch image data. All phase contrast image data was converted to greyscale. For the ‘mixtures’ and MRF method, rather than constructing bivariate mixtures as for multichannel data, the univariate mixture components themselves were used. Since this is also 2D image data, we considered a 4-neighbour MRF although the energy function Eq (1) still has the same form as in the 3D case.

The mouse embryo image data has so far only been used for evaluating cell counting methods [43], however ground truth segmentations of the entire multicellular embryo structures are also provided. Example segmentation output is given in S3 Fig and the mean (and median) F_1_-scores for each segmentation method are given in S1 Table, while F_1_-score line plots are given in S4 Fig. Although the MRF method shows excellent performance, unlike the 3D stack image data from the co-culture model, there may be other segmentation methods that are better suited for the mouse embryo image data. For the Melanoma and Tscratch image data, example output is given in S5 Fig, mean (and median) F_1_-scores are given in S2 Table and corresponding line plots are given in S6 Fig. Here, the MRF segmentation method is compared to three other segmentation methods [44] some of which were specifically designed to segment such ‘wound healing’ assays. However, overall performance is reasonable in comparison and suggests that it may be fruitful to further investigate a MRF based method for such data.

Although there are increasingly more bioimage benchmark data sets becoming available to evaluate a range of computer vision tasks [45], for the most part, these data sets are not applicable for our purposes as they are benchmarks for segmenting (and counting) either single cells or cell nuclei. To the best of our knowledge, we are not aware of any multichannel 3D stack image data available for segmentation benchmarking and we provide such data as supplementary material (S2 and S3 Files). However, we have shown that our segmentation method is not only limited to fluorescence microscopy, but is also more generally applicable to other cellular microscopy image data where the multicellular structures of interest have a different texture to their surrounding environment.

### Model fitting discussion

The local entropy filter is not defined for colour images. However, since we have false colour images it was appropriate to use the local entropy filter on each colour channel separately. We also fit a univariate Gaussian mixture model to each colour channel separately, which would likely not be appropriate for most applications with true colour images. The univariate mixture components were combined to create bivariate densities for each label of interest, which to our knowledge has not previously been considered in a segmentation method. We have shown our segmentation method also performs well on single channel data and in principle our method could be readily extended to cellular microscopy image data with *n* false colour channels. This is the subject of future work.

We assume that each pixel may only be labelled as ‘in focus tumour cells’, ‘in focus CAFs’ or ‘out of focus/background’. Also underlying our approach was the assumption that cells in contact within the same colour channel form part of the same biological structure. Many segmentation methods are concerned with the segmentation of individual cells or cell nuclei even when such objects are in contact [25, 26]. We did not consider separating individual cells as this was not required for the definition of multicellular structures, which was the main focus here. However, if two separate biological structures were in contact within the same colour channel they were likely considered a single structure in the output of our segmentation method. Nevertheless, tumour and CAF structures are segmented as separate objects even if they may be in contact due to the way we have constructed the bivariate densities over both the red and green colour channels.

A three component Gaussian mixture model was fit to both the red and green colour channels of every image in the stack. We found that three mixture components generally fit the pixel density well while also giving an interpretable segmentation output (S7 Fig). A mixture model was fit to each image in the stack as we cannot know beforehand which images may be suitable for model fitting. The fitted mixture model with the greatest sum of paired distances between the means of the mixture components was used to construct the bivariate densities. The same corresponding image in the stack was used to determine the Otsu threshold for both the intensity and local entropy filtered stacks.

For our MRF based method there is a single parameter *β* that was manually set. This parameter value was chosen using a candidate stack of images and the same value was used for all other stacks from the same experiment, which appeared to work well in practice. We found that the range $\frac{1}{100}\leq \beta \leq \frac{1}{10}$ appeared to contain a suitable value of *β* for the image data we considered, preprocessed as it was with the local entropy filter. Smaller values of *β* will result in a ‘rougher’ segmentation whereas larger values of *β* will result in a ‘smoother’ segmentation (S3 Video). Aiming for a certain level of ‘smoothness’ is an appropriate goal for our image data since the objects of interest are expected to be represented as contiguous regions in the images and not as separate individual pixels with no relation to each other. For the IF image data we set $\beta =\frac{1}{40}$, for the LIVE image data we set $\beta =\frac{1}{50}$ and for the FAK image data we set $\beta =\frac{1}{60}$.

One particular aspect of our 3D image data is the relatively large distance between images in the stack. Advantages of this imaging protocol include that it is fast, reduces phototoxicity and photobleaching experienced by the cells, and is hence conducive to high throughput and time course microscopy. We use the standard form of the pairwise potentials for colour image segmentation [33], which takes the distance between neighbouring pixels into account. This means that for the IF and LIVE image data, the two pairwise potentials for neighbouring pixels between images had $\frac{1}{20}$ times the weight of the four pairwise potentials for neighbouring pixels within an image. For the FAK image data, it was $\frac{1}{16}$. Hence, the neighbour information between images does not contribute much to the MRF segmentation and had the image data been captured with even larger distance between images in the stack, this neighbour influence would contribute even less.

An underlying assumption of the MRF model is that relevant neighbouring pixel information, as mediated by the pairwise potentials, is expected to produce a more accurate segmentation. We have shown that including neighbour information significantly improves the segmentation output both quantitatively and qualitatively as compared to using no neighbour information as with the ‘mixtures’ method. As discussed above, owing to the resolution of our 3D image data, the neighbour information between images had relatively small overall influence in the MRF segmentation. If the image data had been captured with a smaller distance between images in the stack, then this influence would have been increased.

To simulate the effect of an increased influence, we can consider the difference in output when setting the pairwise potentials for the within image pixel neighbours to zero (either the vertical or horizontal neighbours within an image). S8 Fig shows that the addition of neighbour information generally appears to increase the segmentation F_1_-score as between the MRF and ‘mixtures’ methods. Hence if image data were to be captured with the same resolution in all dimensions, we would expect that using the neighbour information between images would result in a more accurate segmentation as opposed to not using that information at all. However, comparing segmentation output for image data with different resolutions and drawing such conclusions in general is not applicable as there are likely diminishing returns with additional neighbour information and confounding factors such as setting the parameter *β*.

As discussed above, acquiring 3D image data with a relatively large distance between images in the 3D stack allows for both faster imaging as well as minimises the phototoxicity and photobleaching experienced by the cells. These features are appealing in general but they also indicate that our imaging protocol is conducive to high throughput and time course microscopy. It is possible that ‘time’ could be incorporated within the MRF framework by the addition of two more neighbours for each pixel, ‘forward’ and ‘backward’ in time. The investigation of such applications is the subject of future work.

Note that there may be computational expense issues if the number of images in the 3D stack increases. The largest 3D image stack we considered contained 22 images. The computational expense appears both when fitting the mixture models to each image in the stack as well as for energy minimisation, where every pixel in the entire 3D stack needs to be considered simultaneously.

### Implementation in MATLAB and computational expense

We provide all of the necessary MATLAB code and data to implement our 3D segmentation method (S1 File). The file MRFsegmentation.m provides an implementation of our MRF based method as outlined in Fig 3. By placing all files provided in S1 File on the current path and compiling the code as outlined in README.txt, the user simply needs to be run MRFsegmentation.m to reproduce the segmentation output given in Figs 4 and 12. Extensive comments within the file provide a step by step tutorial of the implementation. All other files are ancillary and are called within MRFsegmentation.m.

Our 3D segmentation method was implemented on a mid 2012 MacBook Pro with a 2.6 GHz Intel Core i7 processor (quad-core) and 16 GB of RAM. Applying the local entropy filter to a 3D stack of images took on average ∼10 seconds and scales linearly with the number of images in the stack. Minimising the energy took on average ∼23 seconds. Fitting a mixture model multiple times to the red and green colour channels of every image in a 3D stack took on average ∼145 seconds. However, fitting the mixture models can be readily implemented in parallel and this process replaces manually choosing the regions for each label as in a standard ‘interactive’ segmentation. Additionally, we found that re-using the same mixture components for the image data from the same experiment worked well in practice and there is no need to continually re-fit the model. This greatly reduces the computational expense when using the method on multiple images from the same data set as in a screening application.

## Conclusions

We have presented an automatic segmentation method based on MRFs for image data from complex organotypic 3D cell culture models that is suitable for the inherent complexity and resolution of the image data, including multiple colour channels and large distances between images in the 3D stack. Our method accurately captures the diverse but equally biologically relevant features of both the multicellular tumour and CAF structures in the 3D stack image data without assuming prior shape information on either type of object. By accurately segmenting the 3D stack of images we are now able to identify important features that were previously elusive, such as investigating the direct contact of tumour cells with CAFs on the surface of tumour organoids in our co-culture model. Such information is fundamentally important when analysing the architecture and tissue-like organisation of 3D cell culture models and in particular the differentiation of tissue architecture that recapitulates the histology of clinical cancers.

Our MRF based method gives a principled way to achieve a multi-label segmentation across multichannel images as well as to obtain ‘smooth’ contiguous segmented regions that may have diverse shapes. Rather than manually seeding regions of the image, our method only requires a single parameter *β* to be manually determined and we found that it is reasonable to use the same value across all images from the same experiment. We evaluated segmentation performance and showed both that our method performs well, and is also more widely applicable to other types of cellular microscopy data, not just limited to fluorescence microscopy.

## Supporting Information

S1 Video Video of example 3D segmentation output corresponding to Fig 4. (AVI)

S2 Video Video of example 3D segmentation output corresponding to Fig 12. (AVI)

S3 Video Video of example 3D segmentation output where the value of *β* is varied. (AVI)

S1 Files MATLAB code. The MATLAB code to obtain the segmentation output presented in this paper.
(ZIP)

S2 Files IF image data and ground truth labels. (ZIP)

S3 Files LIVE image data and ground truth labels. (ZIP)

S1 Fig Influence of the MRF parameters. Example ‘ground truth’ image and noisy version obtained by adding independent white noise to each pixel value. The MRF segmentation output demonstrates the trade-off between correspondence and smoothness that is obtained through different values of *β*.
(TIFF)

S2 Fig Maximum intensity projections of the FAK image data. The two blue asterisks indicate the two image stacks that have particularly high total volume, total surface area and total tumour-stroma contact in Fig 5.
(TIFF)

S3 Fig Example segmentation output for the mouse embryo image data. (TIFF)

S4 Fig Box and line plots of the of F_1_-scores for the mouse embryo image data. (TIFF)

S5 Fig Example segmentation output for the Melanoma and Tscratch image data. (TIFF)

S6 Fig Box and line plots of the F_1_-scores for the MRF segmentation method and of the F_1_-scores previously calculated for three other segmentation methods [44] for the Melanoma and Tscratch image data. (TIFF)

S7 Fig Fitting a mixture model to the local entropy filtered image. Example red and green channel images from a 3D stack with the corresponding local entropy filtered versions. The histograms of the local entropy filtered pixel values are overlaid with the three estimated mixture components.
(TIFF)

S8 Fig Comparison between segmentation output for the IF image data based on different neighbour information. F_1_-scores for the MRF segmentation (negligible neighbour influence between images), the MRF segmentation with only the horizontal neighbours within images (a similar result is obtained using just the vertical neighbours) and the ‘mixtures’ segmentation (no neighbour influence at all).
(TIFF)

S1 Table Mean (and median) macro F_1_-scores of the segmentation methods for the mouse embryo image data. (PDF)

S2 Table Mean (and median) F_1_-scores of the MRF segmentation method, and mean (and median) F_1_-scores previously calculated for three other segmentation methods [44] for the Melanoma and Tscratch image data. (PDF)
