# Supplementary figures and images for: Segmentation of Image Data from Complex Organotypic 3D Models of Cancer Tissues with Markov Random Fields
Source: PLoS One. 2015 Dec 2;10(12):e0143798. doi: 10.1371/journal.pone.0143798 (PMC4668034; doi:10.1371/journal.pone.0143798)

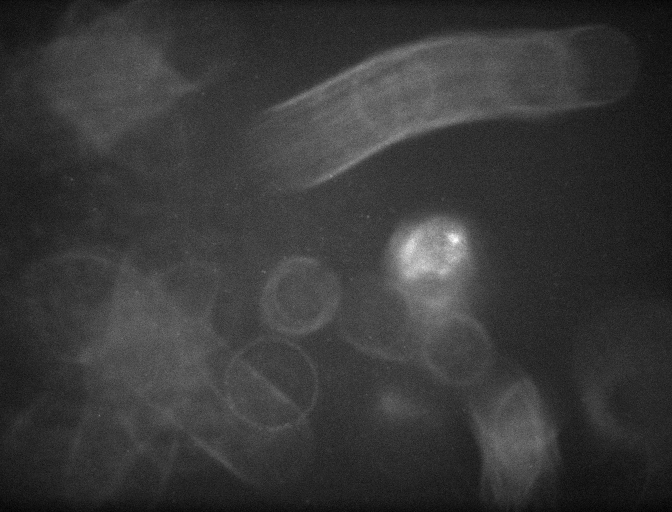

Supplement: S1 Files — The MATLAB code to obtain the segmentation output presented in this paper. (ZIP) [file pone.0143798.s004.zip › code/fig12CAFs.tif]

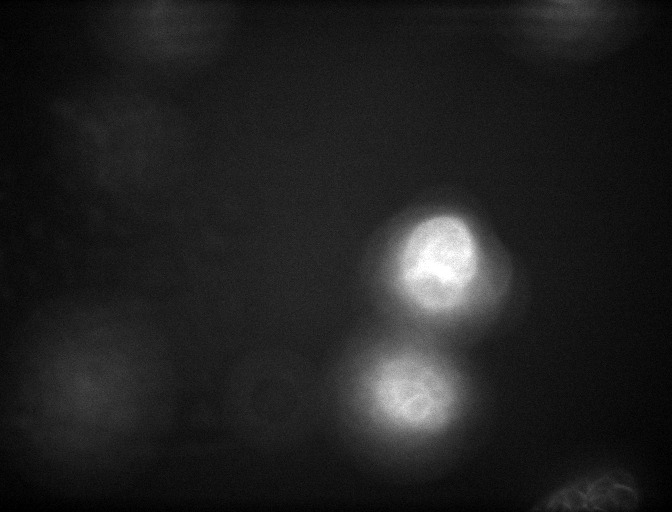

Supplement: S1 Files — The MATLAB code to obtain the segmentation output presented in this paper. (ZIP) [file pone.0143798.s004.zip › code/fig12tumourcells.tif]

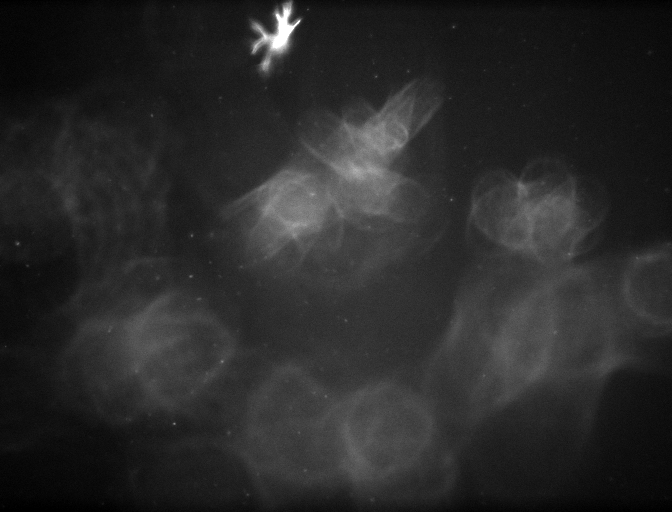

Supplement: S1 Files — The MATLAB code to obtain the segmentation output presented in this paper. (ZIP) [file pone.0143798.s004.zip › code/fig4CAFs.tif]

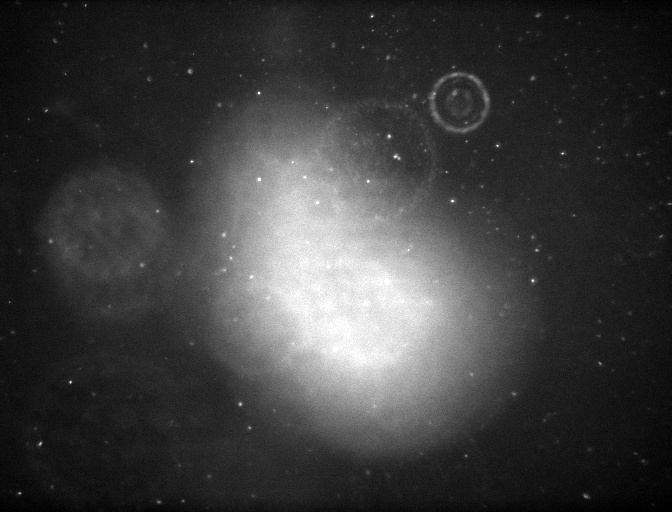

Supplement: S1 Files — The MATLAB code to obtain the segmentation output presented in this paper. (ZIP) [file pone.0143798.s004.zip › code/fig4tumourcells.tif]

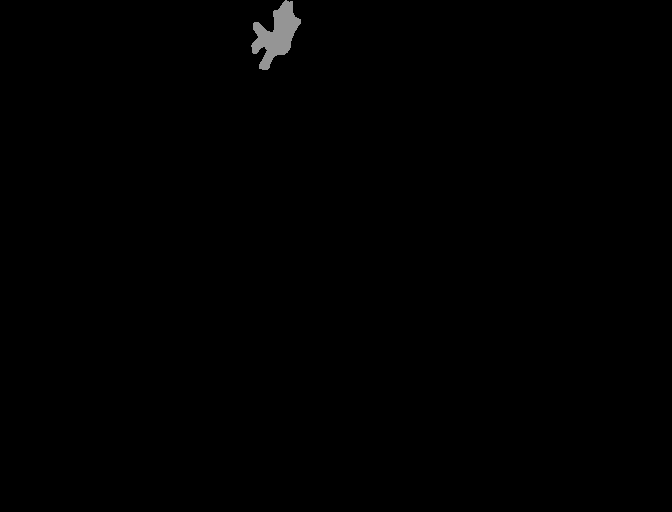

Supplement: S2 Files — (ZIP) [file pone.0143798.s005.zip › IFimagedata/IFdata1labels.tif]

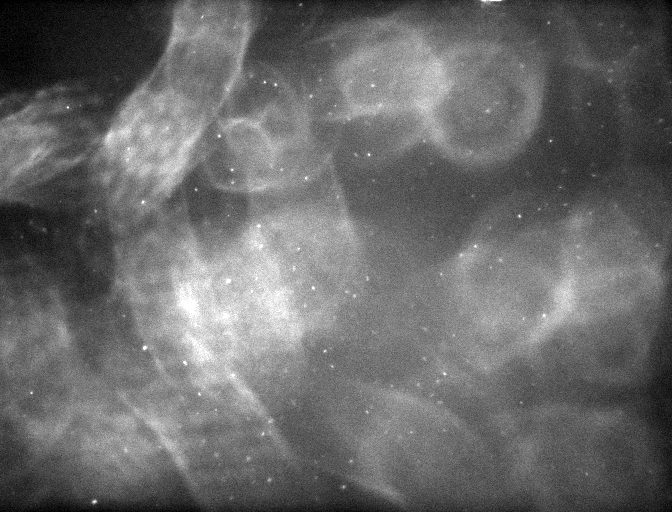

Supplement: S2 Files — (ZIP) [file pone.0143798.s005.zip › IFimagedata/IFdata2green.tif]

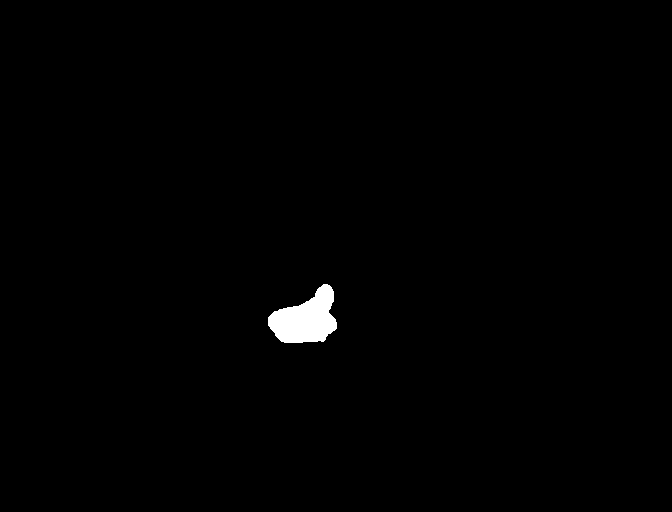

Supplement: S2 Files — (ZIP) [file pone.0143798.s005.zip › IFimagedata/IFdata2labels.tif]

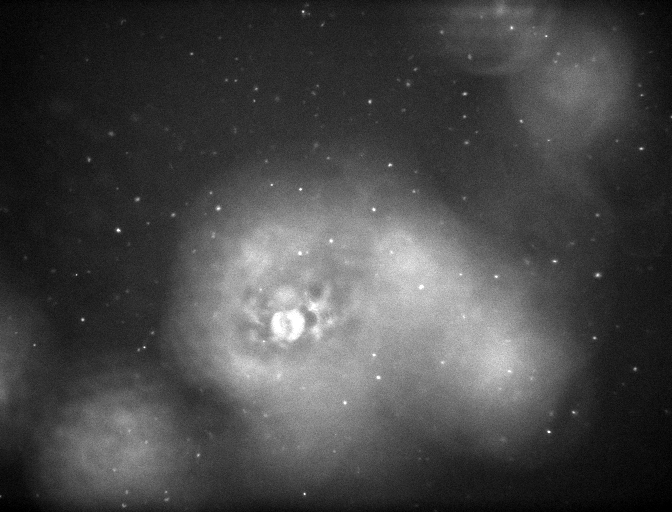

Supplement: S2 Files — (ZIP) [file pone.0143798.s005.zip › IFimagedata/IFdata2red.tif]

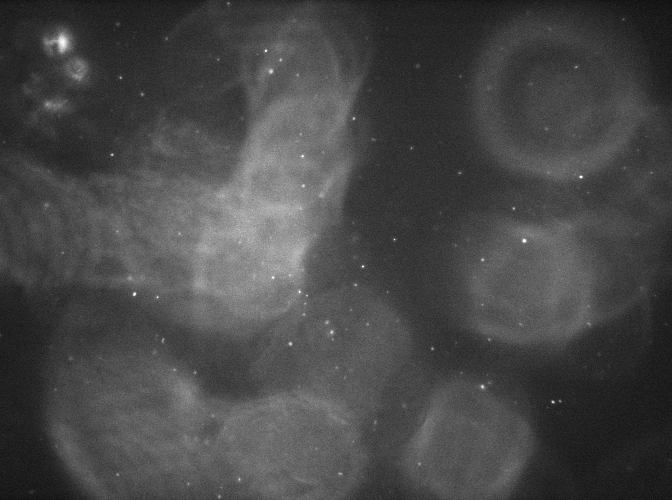

Supplement: S2 Files — (ZIP) [file pone.0143798.s005.zip › IFimagedata/IFdata3green.tif]

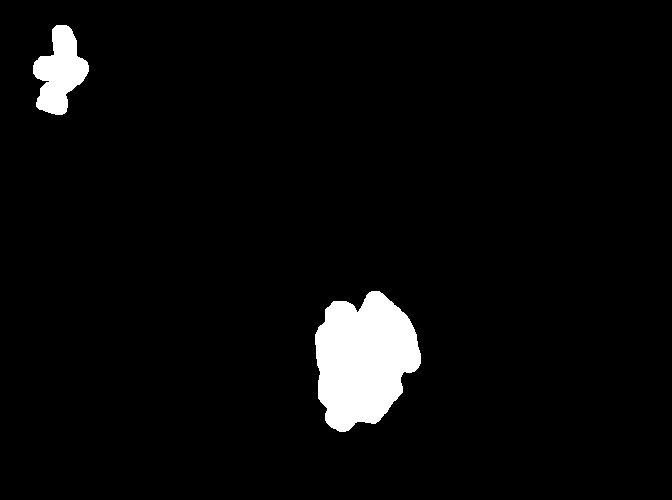

Supplement: S2 Files — (ZIP) [file pone.0143798.s005.zip › IFimagedata/IFdata3labels.tif]

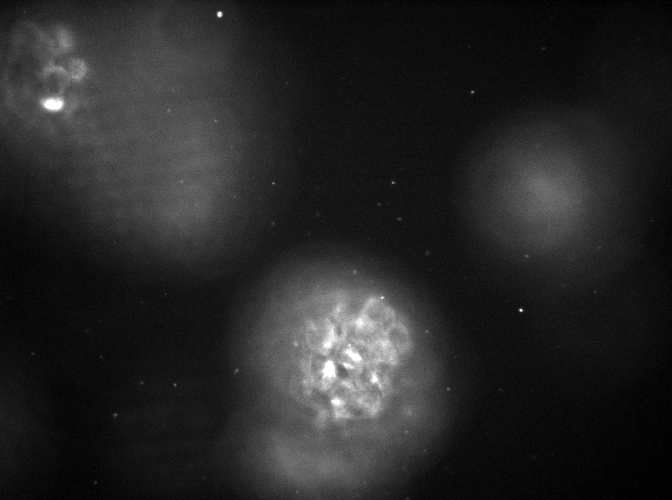

Supplement: S2 Files — (ZIP) [file pone.0143798.s005.zip › IFimagedata/IFdata3red.tif]

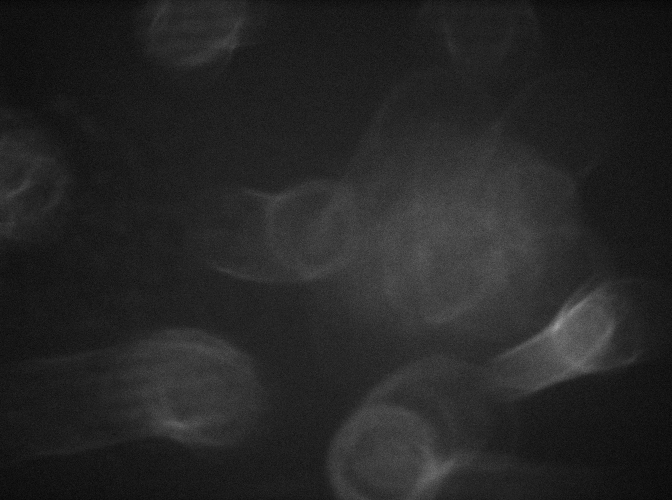

Supplement: S2 Files — (ZIP) [file pone.0143798.s005.zip › IFimagedata/IFdata4green.tif]

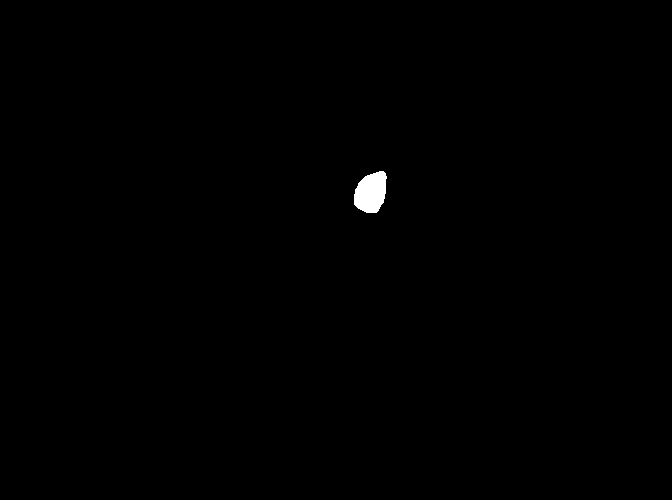

Supplement: S2 Files — (ZIP) [file pone.0143798.s005.zip › IFimagedata/IFdata4labels.tif]

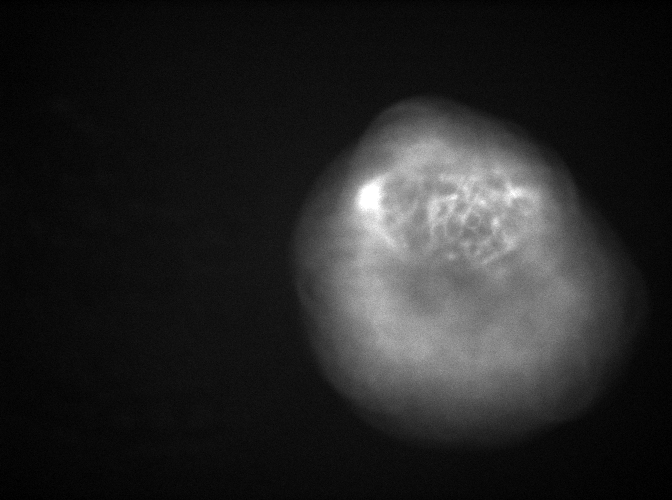

Supplement: S2 Files — (ZIP) [file pone.0143798.s005.zip › IFimagedata/IFdata4red.tif]

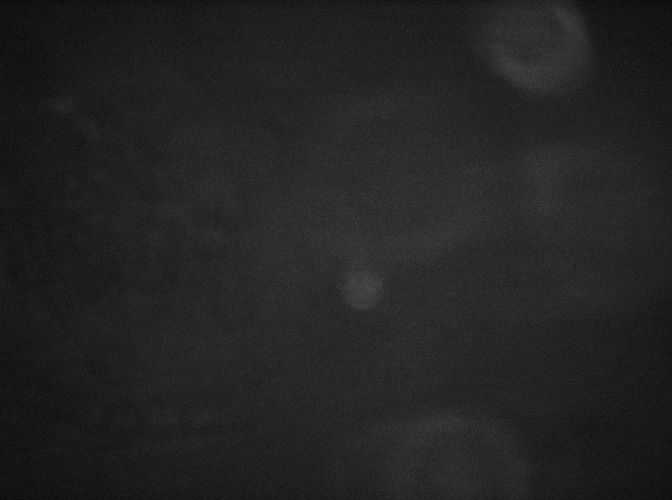

Supplement: S2 Files — (ZIP) [file pone.0143798.s005.zip › IFimagedata/IFdata5green.tif]

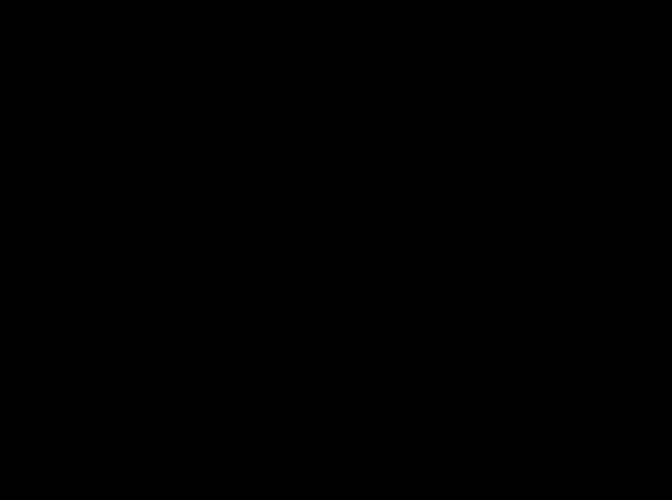

Supplement: S2 Files — (ZIP) [file pone.0143798.s005.zip › IFimagedata/IFdata5labels.tif]

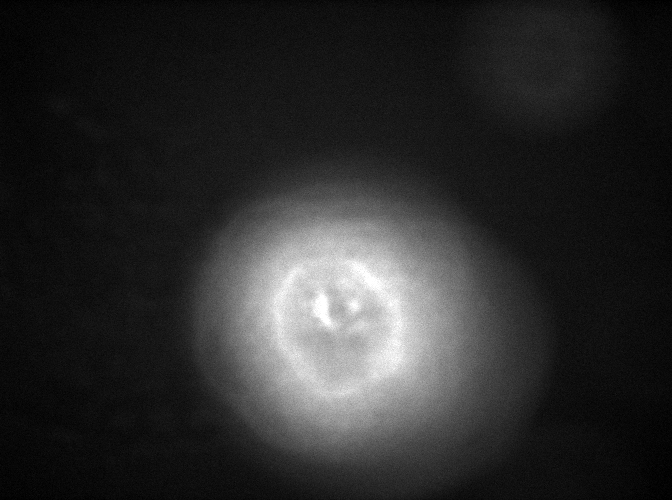

Supplement: S2 Files — (ZIP) [file pone.0143798.s005.zip › IFimagedata/IFdata5red.tif]

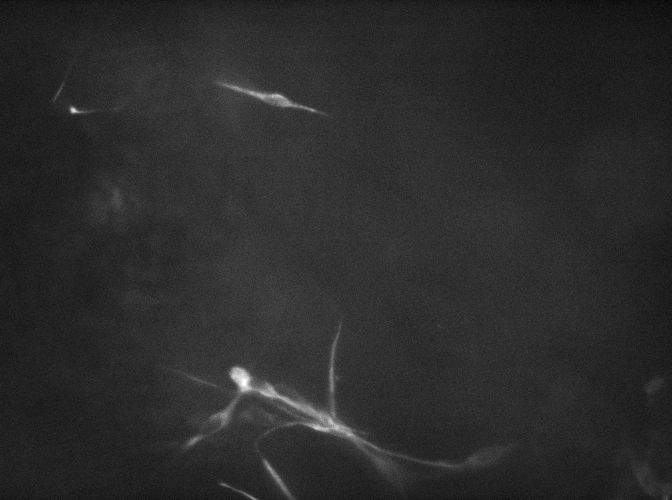

Supplement: S2 Files — (ZIP) [file pone.0143798.s005.zip › IFimagedata/IFdata6green.tif]

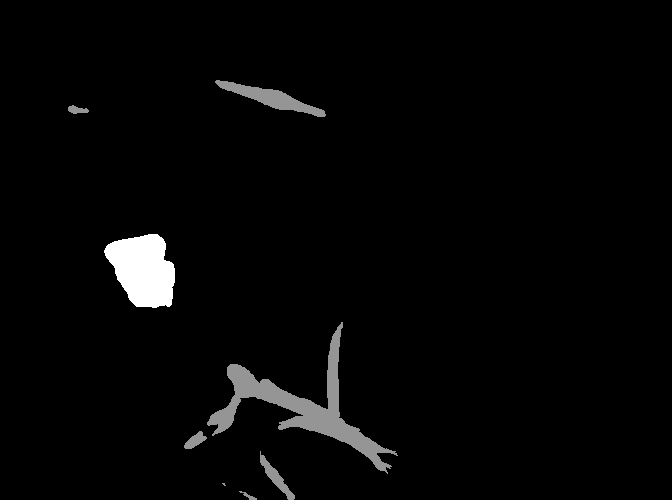

Supplement: S2 Files — (ZIP) [file pone.0143798.s005.zip › IFimagedata/IFdata6labels.tif]

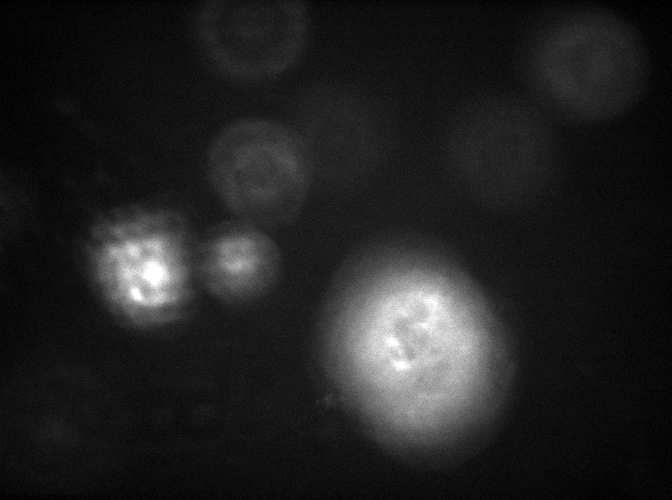

Supplement: S2 Files — (ZIP) [file pone.0143798.s005.zip › IFimagedata/IFdata6red.tif]

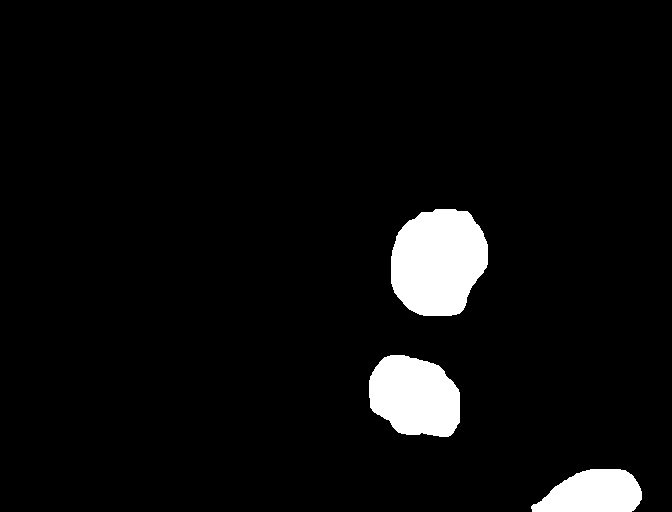

Supplement: S2 Files — (ZIP) [file pone.0143798.s005.zip › IFimagedata/IFdata7labels.tif]

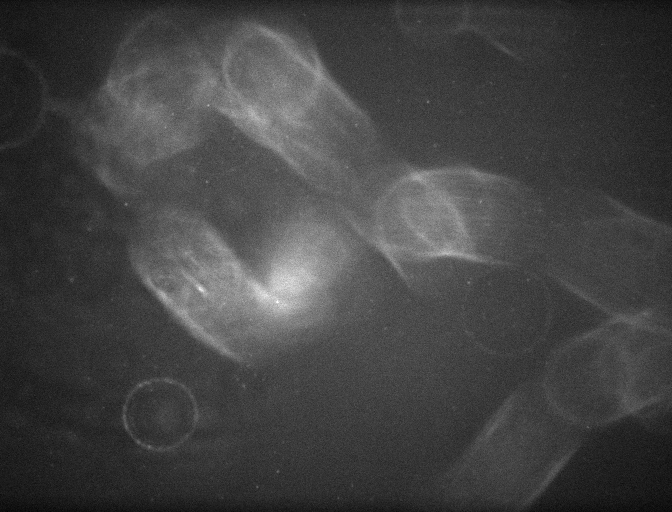

Supplement: S2 Files — (ZIP) [file pone.0143798.s005.zip › IFimagedata/IFdata8green.tif]

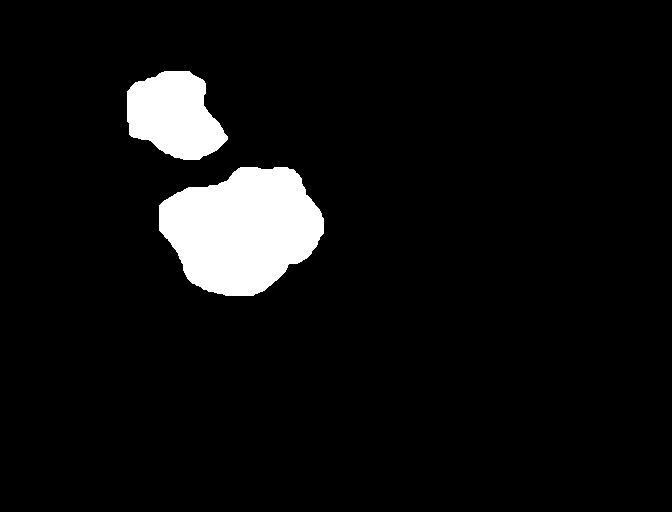

Supplement: S2 Files — (ZIP) [file pone.0143798.s005.zip › IFimagedata/IFdata8labels.tif]

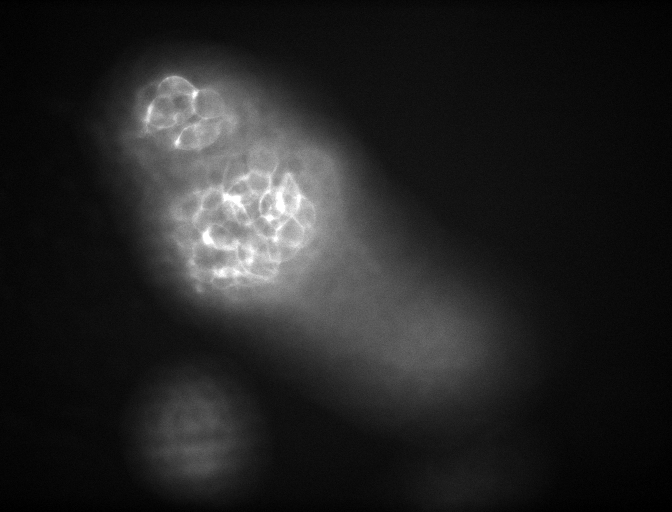

Supplement: S2 Files — (ZIP) [file pone.0143798.s005.zip › IFimagedata/IFdata8red.tif]

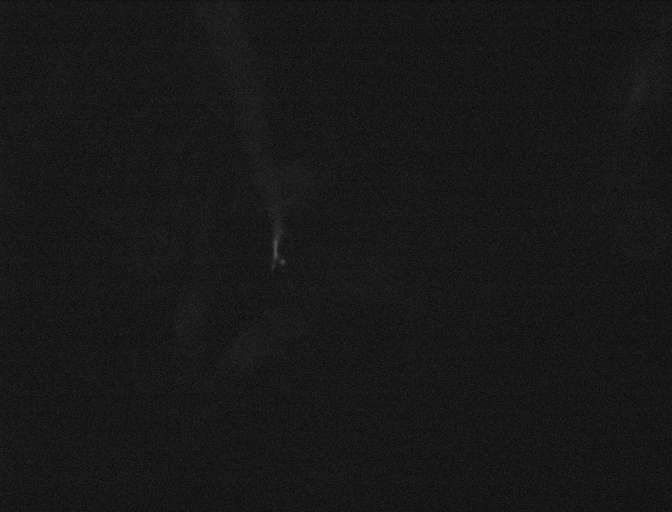

Supplement: S3 Files — (ZIP) [file pone.0143798.s006.zip › LIVEimagedata/LIVEdata10green.tif]

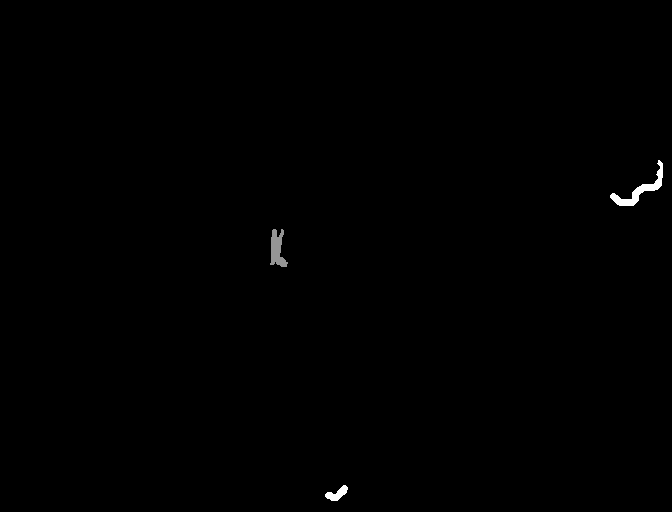

Supplement: S3 Files — (ZIP) [file pone.0143798.s006.zip › LIVEimagedata/LIVEdata10labels.tif]

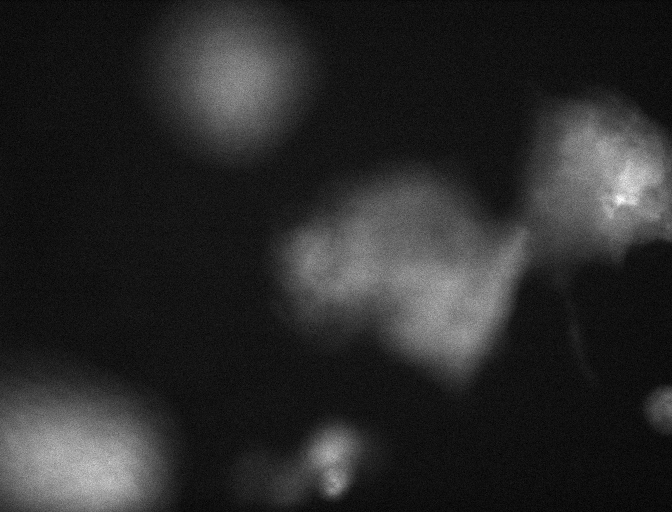

Supplement: S3 Files — (ZIP) [file pone.0143798.s006.zip › LIVEimagedata/LIVEdata10red.tif]

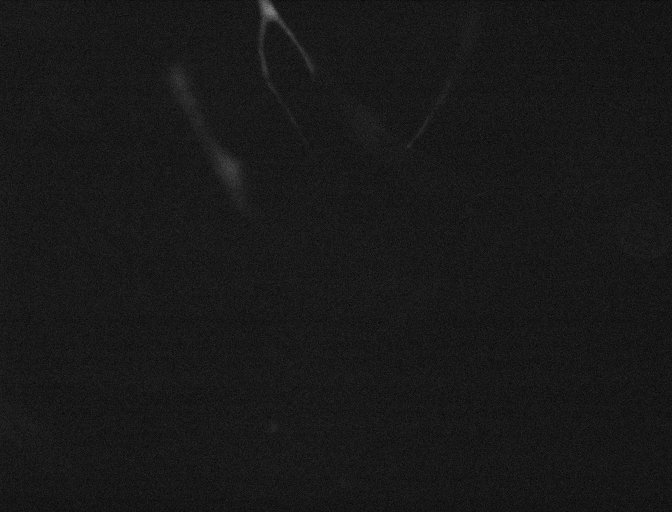

Supplement: S3 Files — (ZIP) [file pone.0143798.s006.zip › LIVEimagedata/LIVEdata11green.tif]

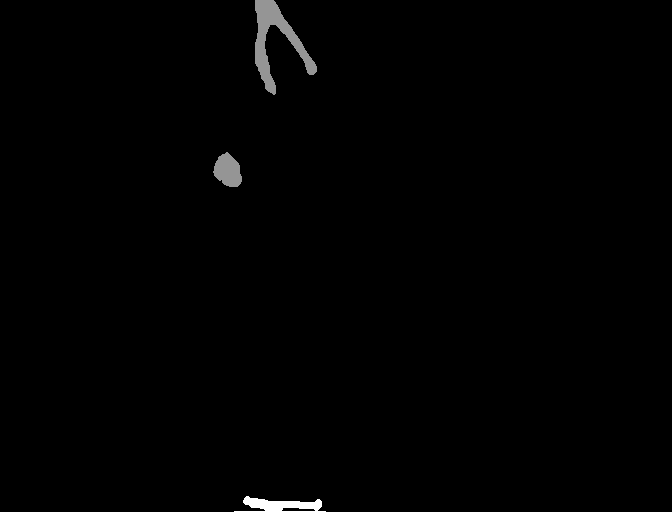

Supplement: S3 Files — (ZIP) [file pone.0143798.s006.zip › LIVEimagedata/LIVEdata11labels.tif]

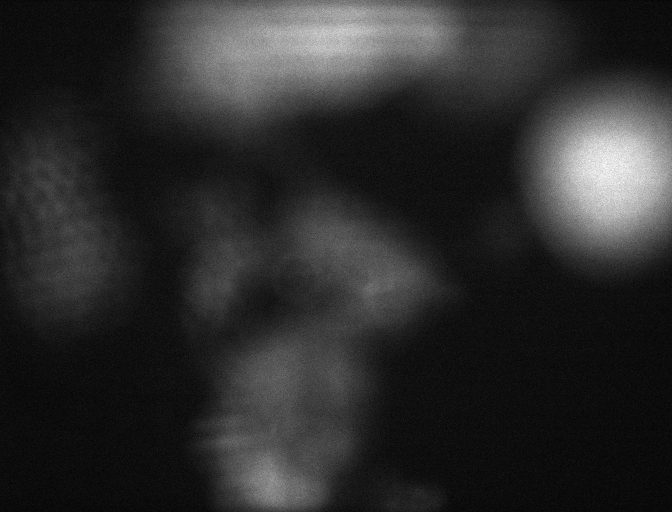

Supplement: S3 Files — (ZIP) [file pone.0143798.s006.zip › LIVEimagedata/LIVEdata11red.tif]

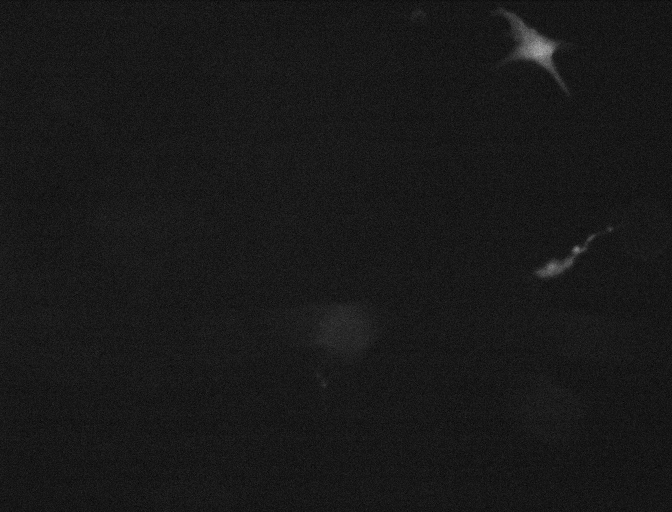

Supplement: S3 Files — (ZIP) [file pone.0143798.s006.zip › LIVEimagedata/LIVEdata12green.tif]

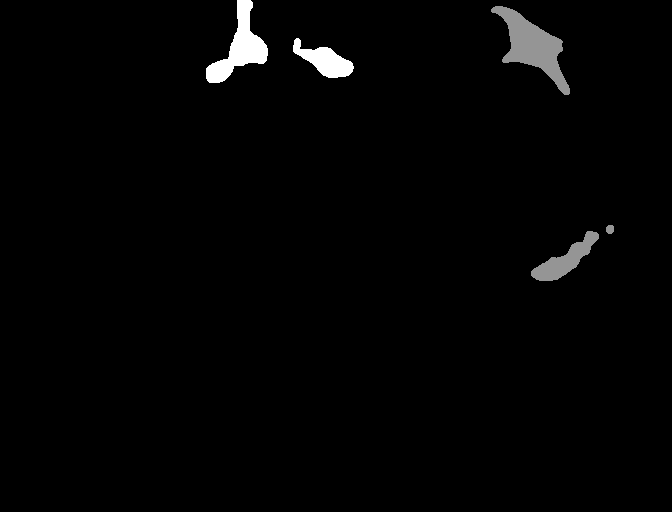

Supplement: S3 Files — (ZIP) [file pone.0143798.s006.zip › LIVEimagedata/LIVEdata12labels.tif]

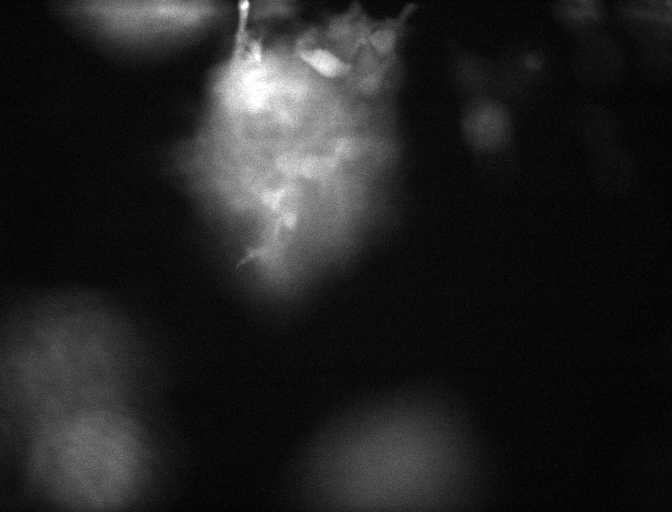

Supplement: S3 Files — (ZIP) [file pone.0143798.s006.zip › LIVEimagedata/LIVEdata12red.tif]

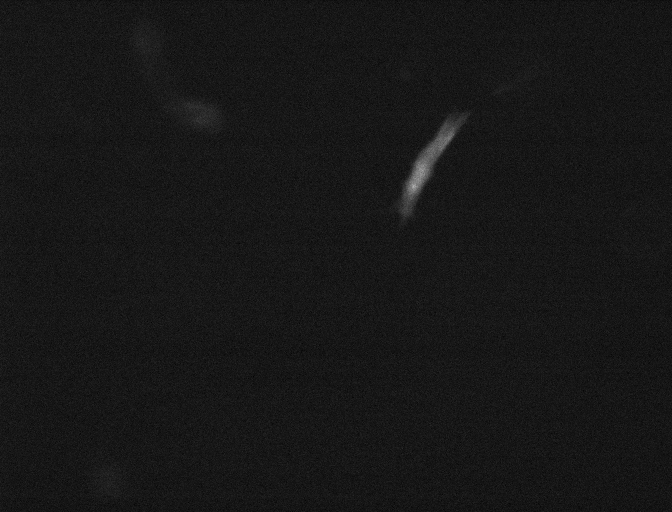

Supplement: S3 Files — (ZIP) [file pone.0143798.s006.zip › LIVEimagedata/LIVEdata1green.tif]

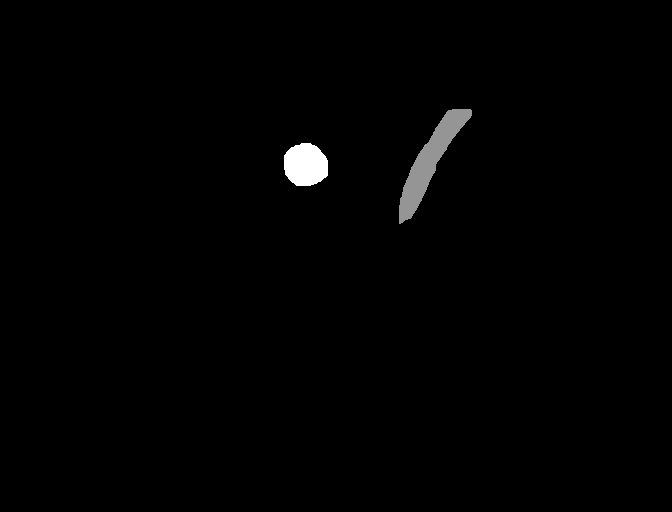

Supplement: S3 Files — (ZIP) [file pone.0143798.s006.zip › LIVEimagedata/LIVEdata1labels.tif]

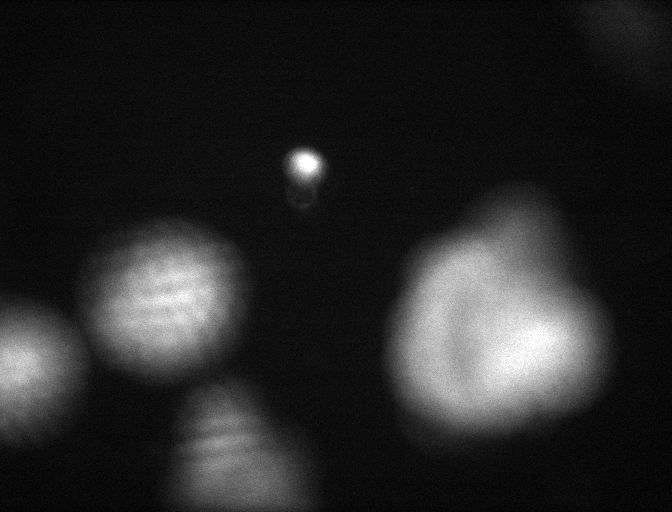

Supplement: S3 Files — (ZIP) [file pone.0143798.s006.zip › LIVEimagedata/LIVEdata1red.tif]

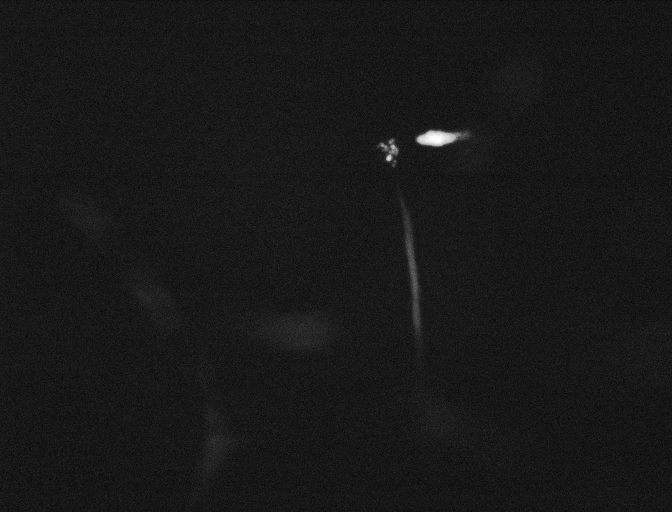

Supplement: S3 Files — (ZIP) [file pone.0143798.s006.zip › LIVEimagedata/LIVEdata2green.tif]

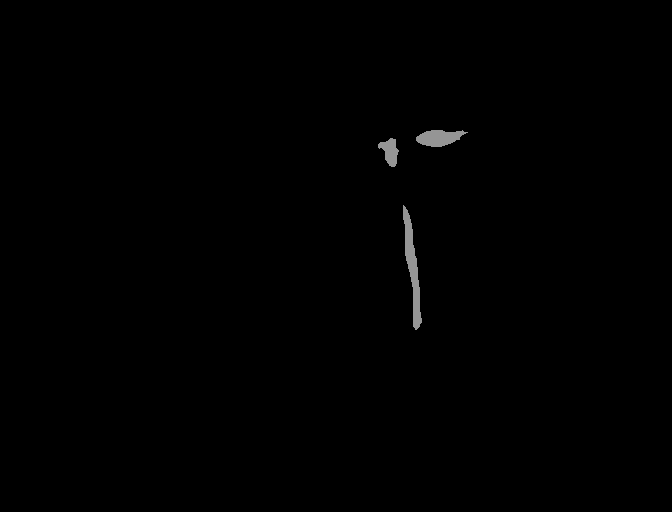

Supplement: S3 Files — (ZIP) [file pone.0143798.s006.zip › LIVEimagedata/LIVEdata2labels.tif]

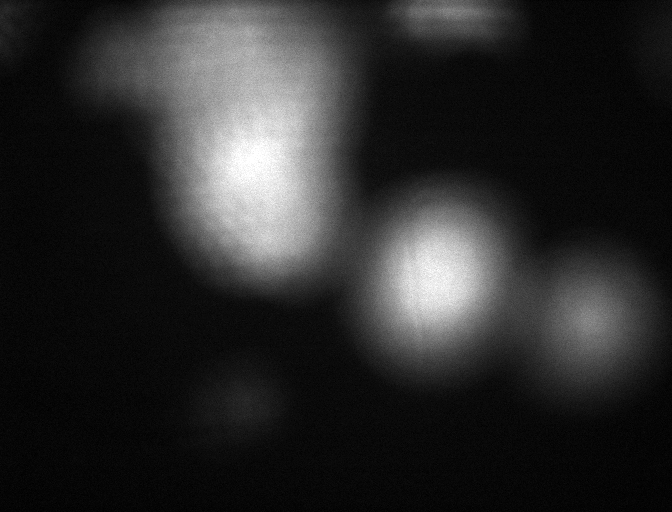

Supplement: S3 Files — (ZIP) [file pone.0143798.s006.zip › LIVEimagedata/LIVEdata2red.tif]

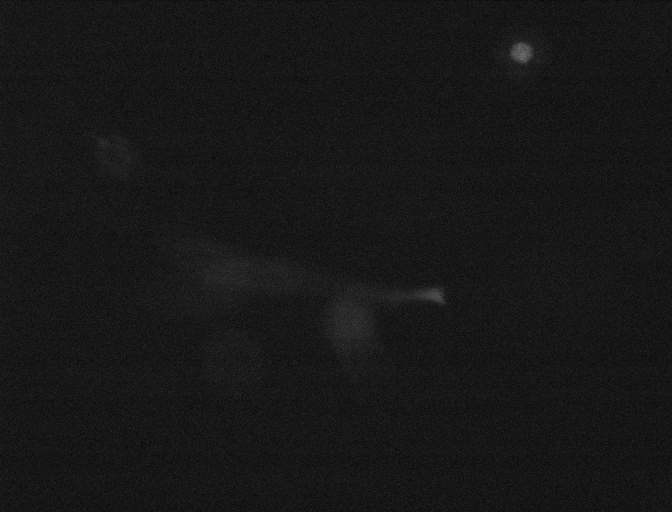

Supplement: S3 Files — (ZIP) [file pone.0143798.s006.zip › LIVEimagedata/LIVEdata3green.tif]

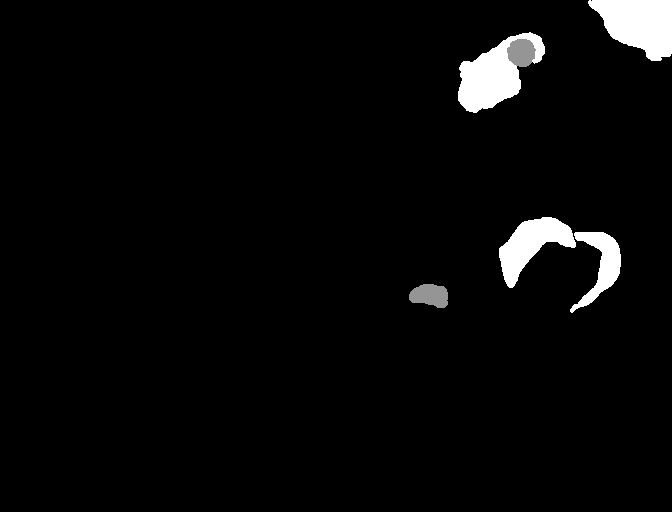

Supplement: S3 Files — (ZIP) [file pone.0143798.s006.zip › LIVEimagedata/LIVEdata3labels.tif]

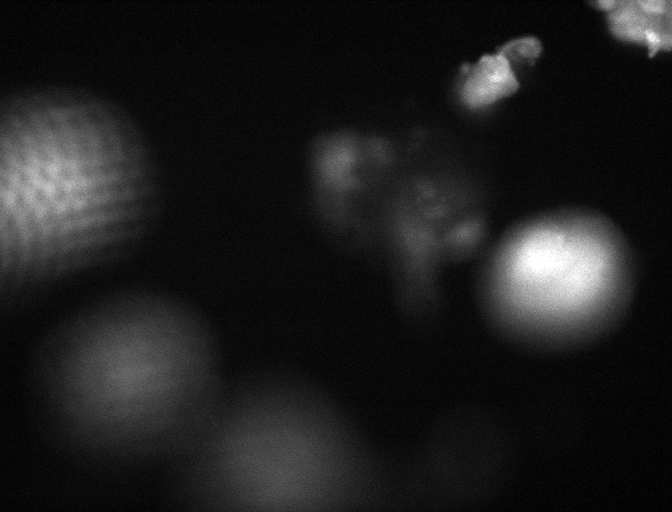

Supplement: S3 Files — (ZIP) [file pone.0143798.s006.zip › LIVEimagedata/LIVEdata3red.tif]

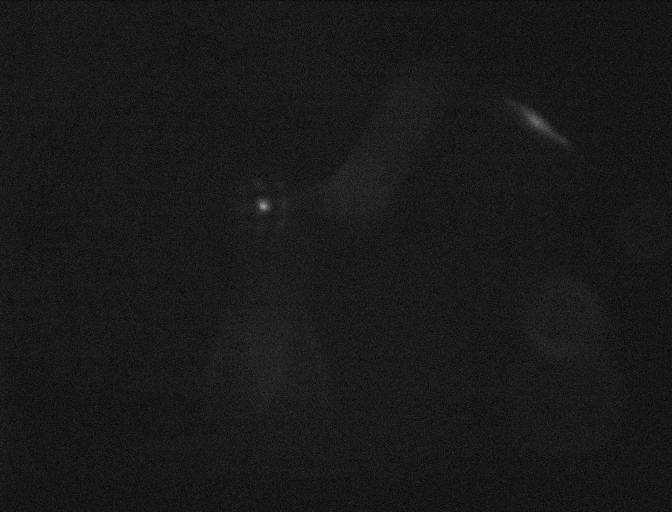

Supplement: S3 Files — (ZIP) [file pone.0143798.s006.zip › LIVEimagedata/LIVEdata4green.tif]

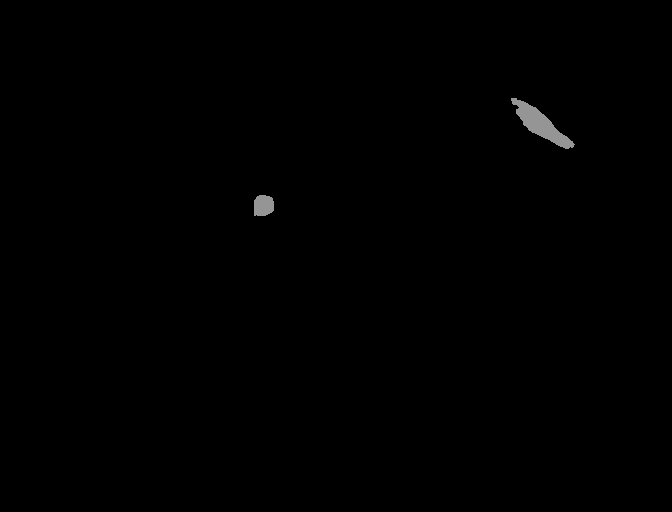

Supplement: S3 Files — (ZIP) [file pone.0143798.s006.zip › LIVEimagedata/LIVEdata4labels.tif]

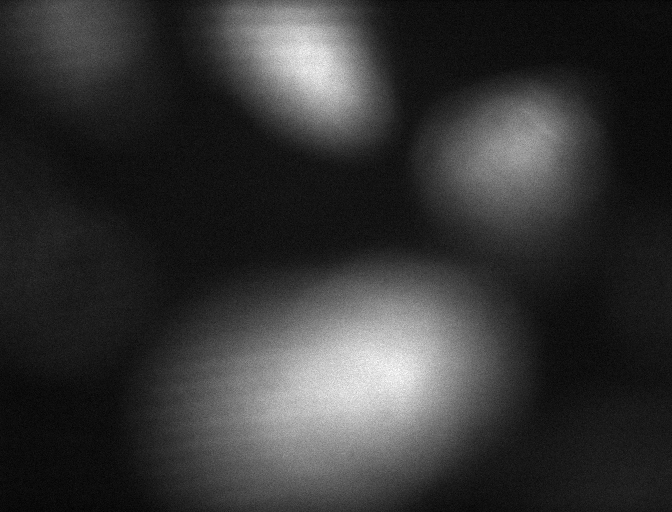

Supplement: S3 Files — (ZIP) [file pone.0143798.s006.zip › LIVEimagedata/LIVEdata4red.tif]

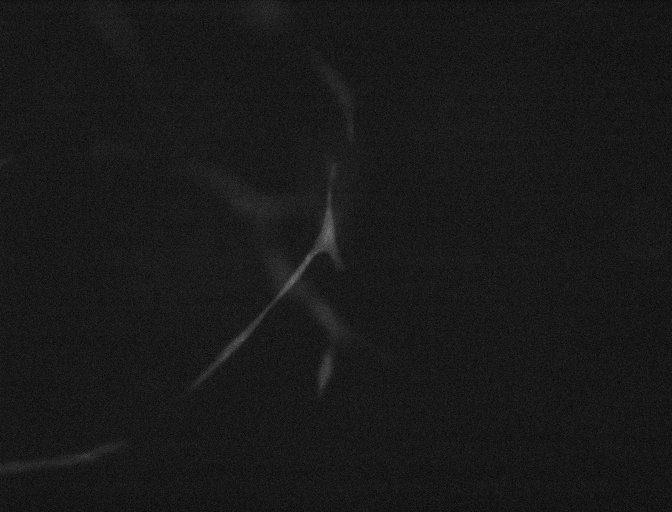

Supplement: S3 Files — (ZIP) [file pone.0143798.s006.zip › LIVEimagedata/LIVEdata5green.tif]

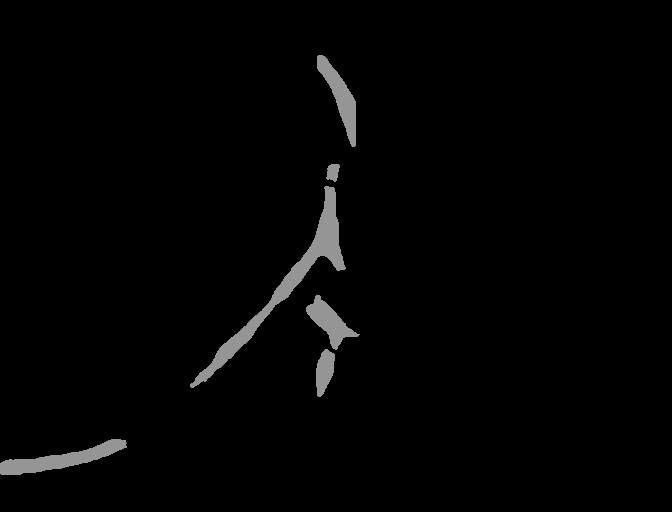

Supplement: S3 Files — (ZIP) [file pone.0143798.s006.zip › LIVEimagedata/LIVEdata5labels.tif]

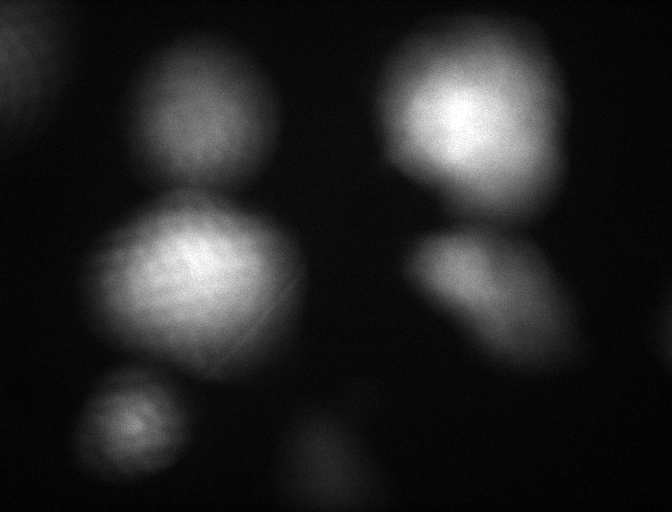

Supplement: S3 Files — (ZIP) [file pone.0143798.s006.zip › LIVEimagedata/LIVEdata5red.tif]

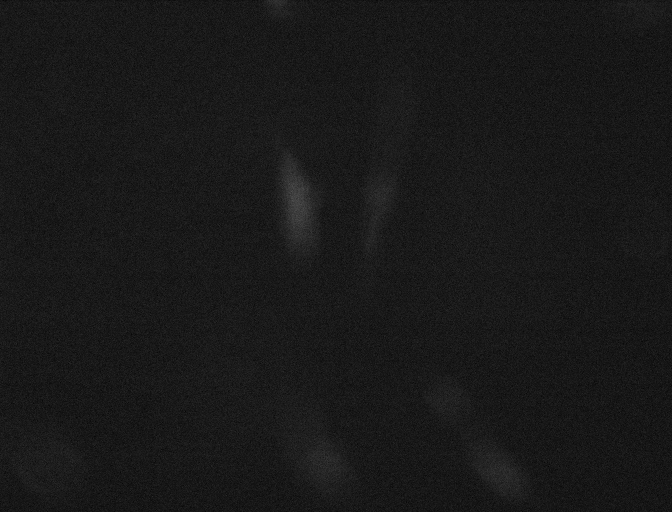

Supplement: S3 Files — (ZIP) [file pone.0143798.s006.zip › LIVEimagedata/LIVEdata6green.tif]

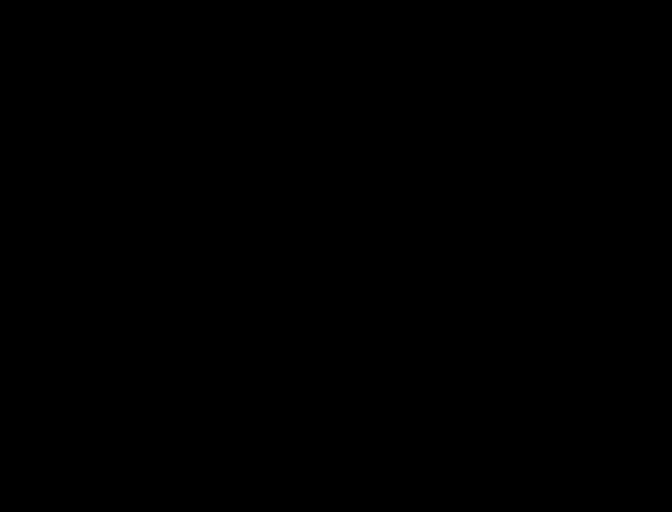

Supplement: S3 Files — (ZIP) [file pone.0143798.s006.zip › LIVEimagedata/LIVEdata6labels.tif]

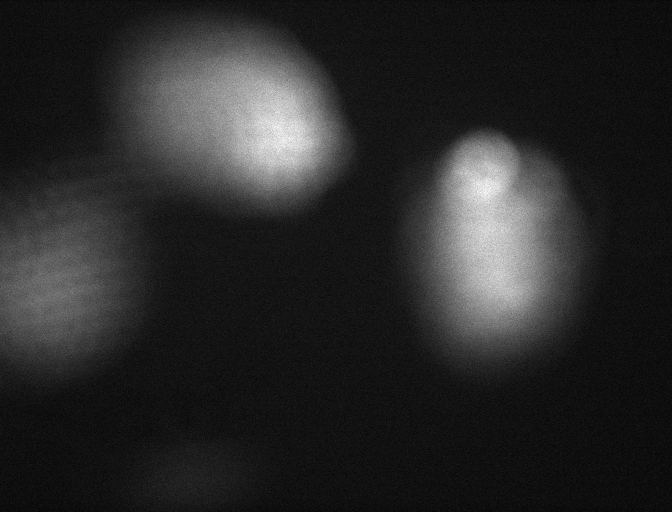

Supplement: S3 Files — (ZIP) [file pone.0143798.s006.zip › LIVEimagedata/LIVEdata6red.tif]

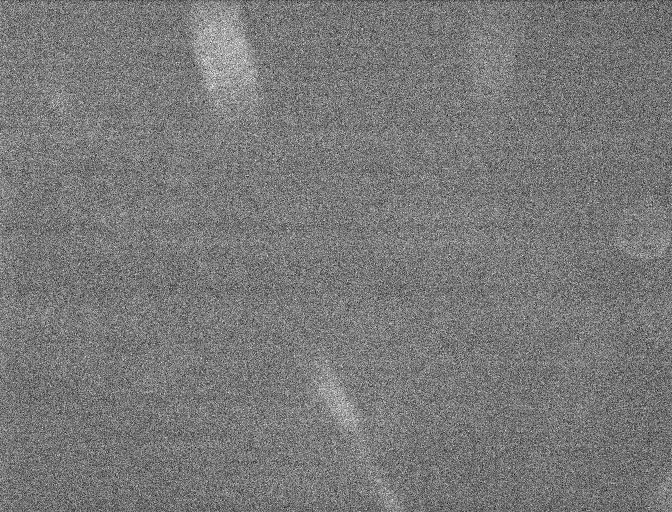

Supplement: S3 Files — (ZIP) [file pone.0143798.s006.zip › LIVEimagedata/LIVEdata7green.tif]

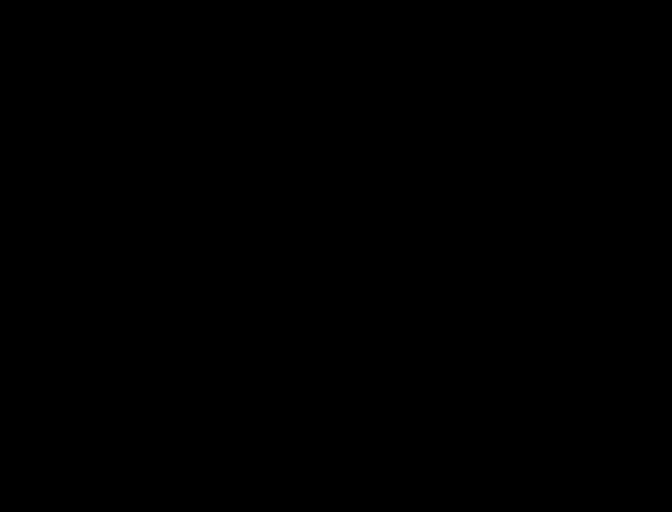

Supplement: S3 Files — (ZIP) [file pone.0143798.s006.zip › LIVEimagedata/LIVEdata7labels.tif]

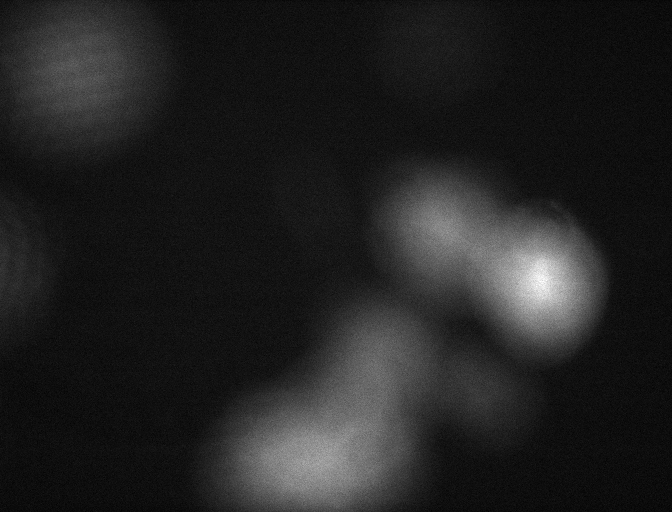

Supplement: S3 Files — (ZIP) [file pone.0143798.s006.zip › LIVEimagedata/LIVEdata7red.tif]

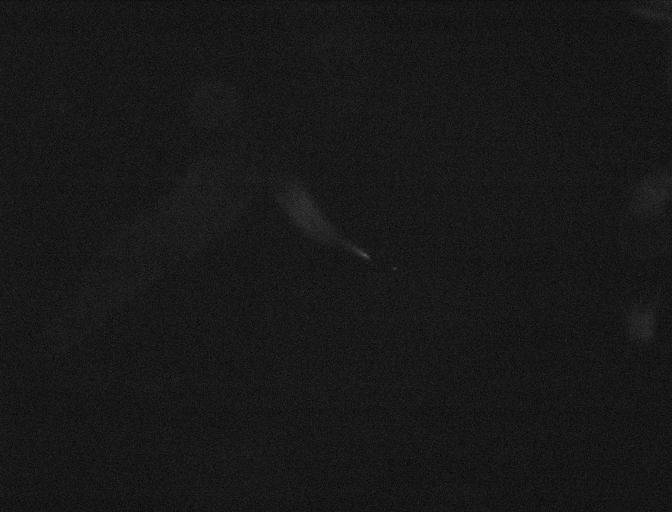

Supplement: S3 Files — (ZIP) [file pone.0143798.s006.zip › LIVEimagedata/LIVEdata8green.tif]

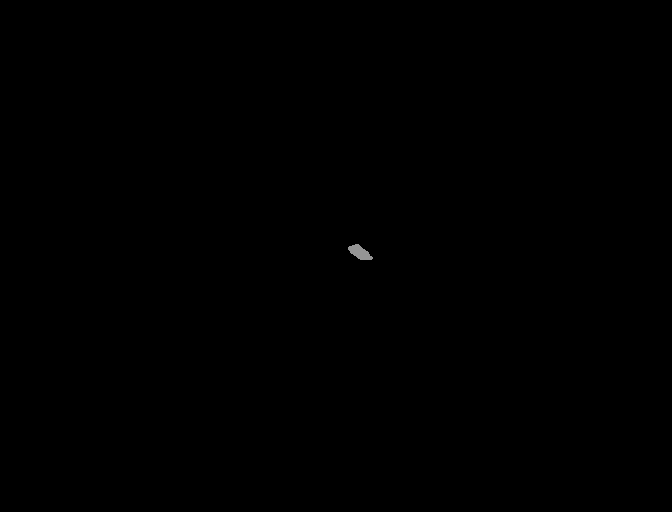

Supplement: S3 Files — (ZIP) [file pone.0143798.s006.zip › LIVEimagedata/LIVEdata8labels.tif]

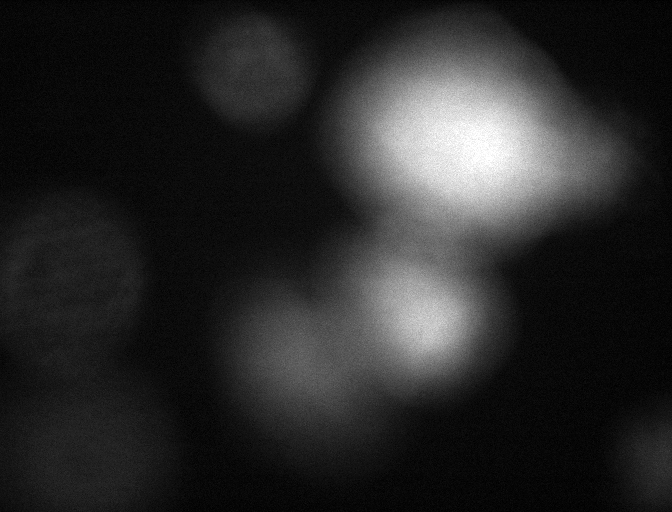

Supplement: S3 Files — (ZIP) [file pone.0143798.s006.zip › LIVEimagedata/LIVEdata8red.tif]

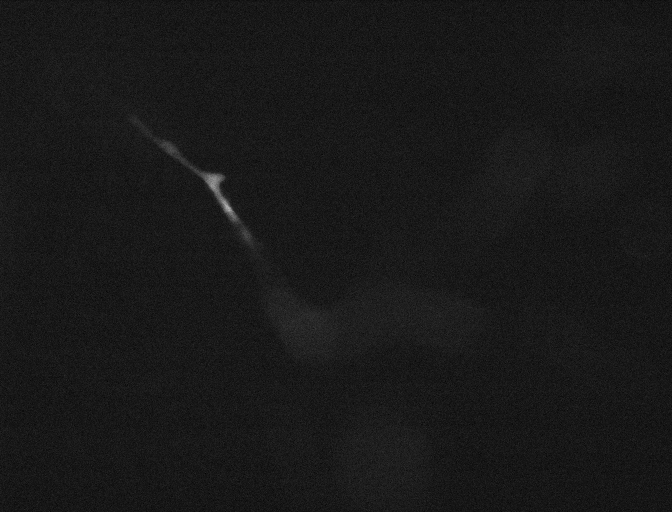

Supplement: S3 Files — (ZIP) [file pone.0143798.s006.zip › LIVEimagedata/LIVEdata9green.tif]

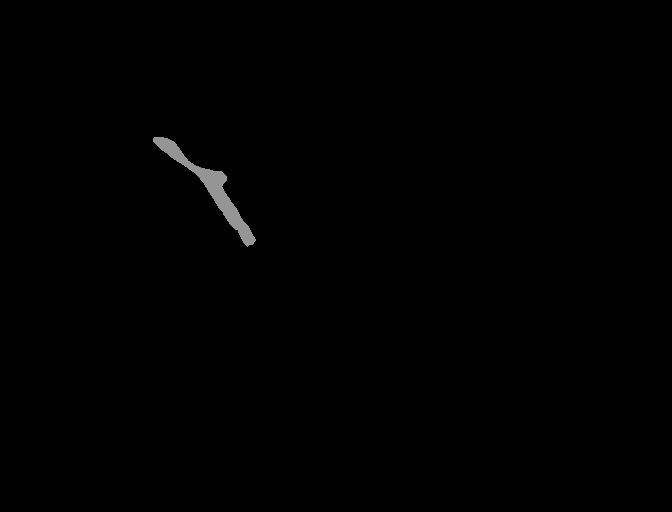

Supplement: S3 Files — (ZIP) [file pone.0143798.s006.zip › LIVEimagedata/LIVEdata9labels.tif]

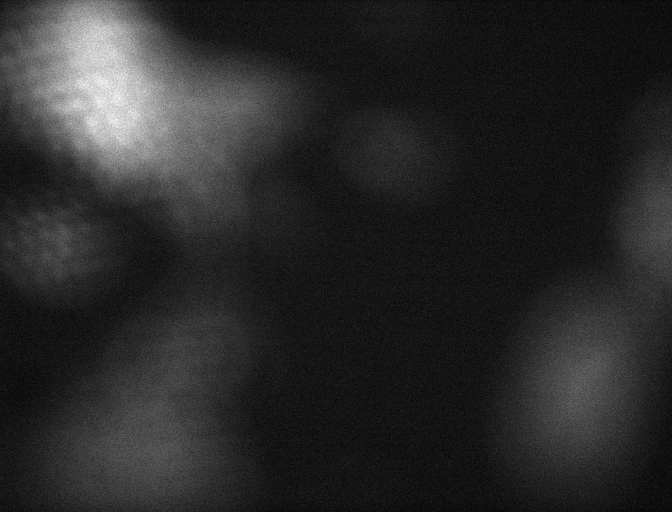

Supplement: S3 Files — (ZIP) [file pone.0143798.s006.zip › LIVEimagedata/LIVEdata9red.tif]

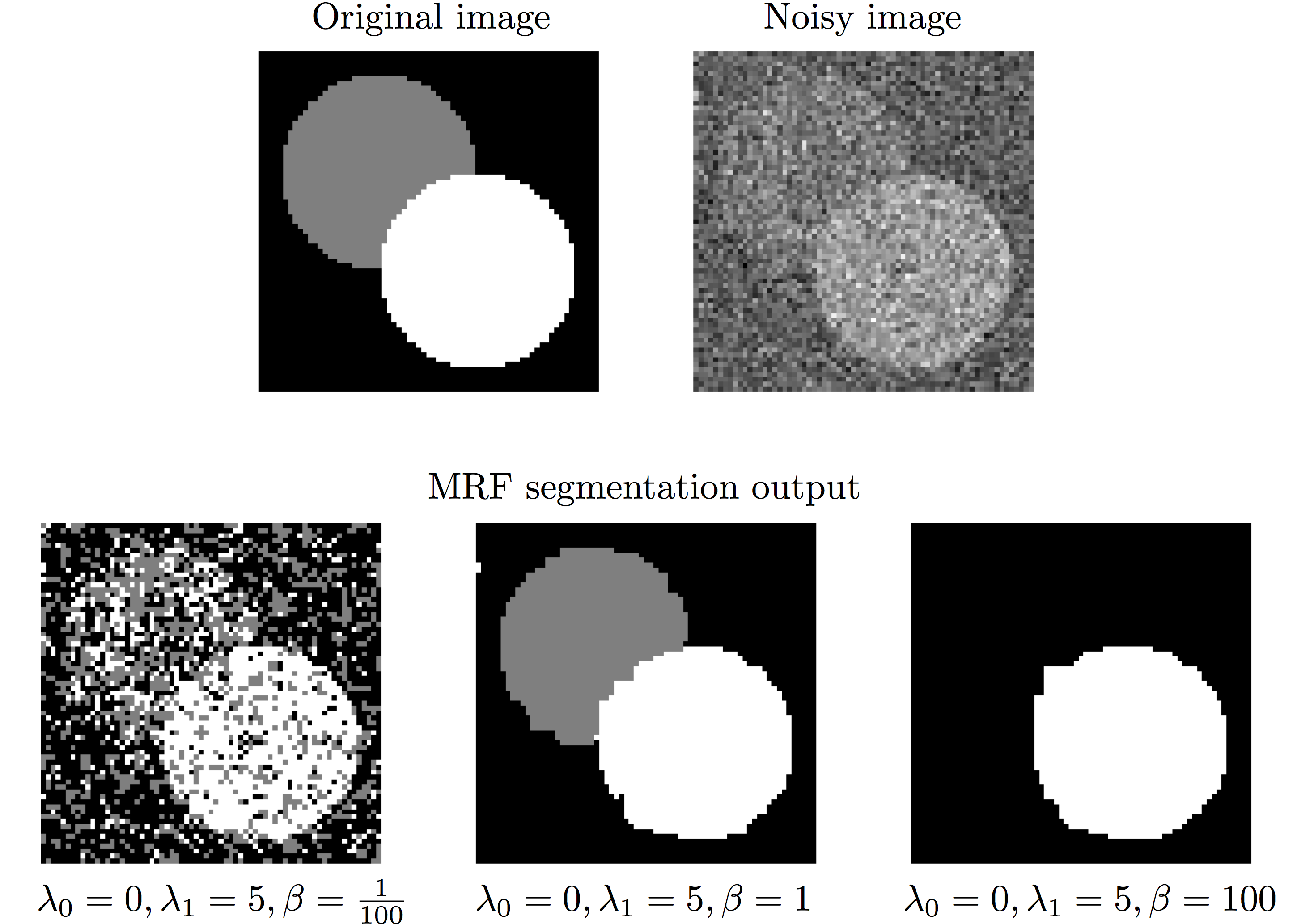

Supplement: S1 Fig — Example ‘ground truth’ image and noisy version obtained by adding independent white noise to each pixel value. The MRF segmentation output demonstrates the trade-off between correspondence and smoothness that is obtained through different values of β. (TIFF) [file pone.0143798.s007.tiff]

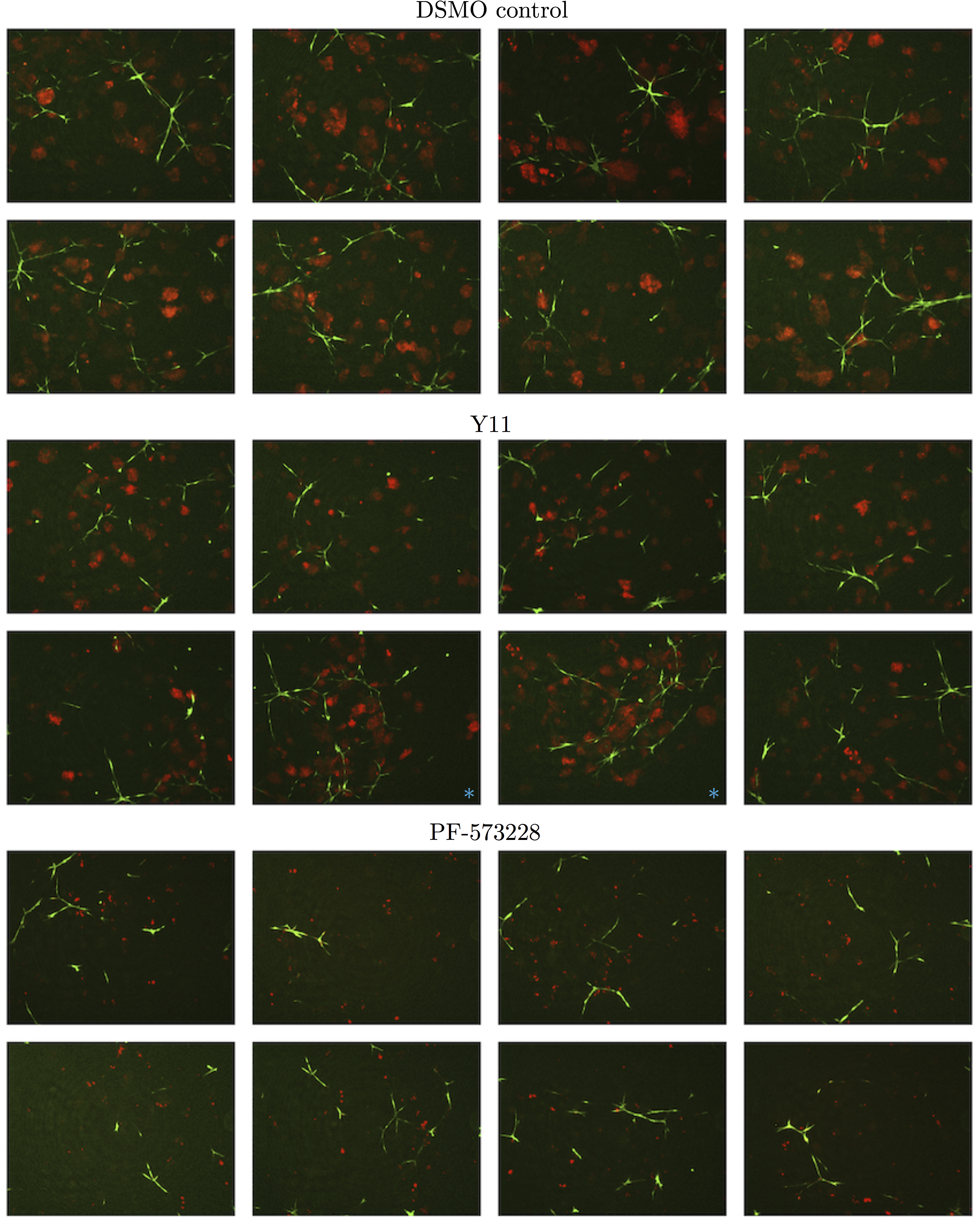

Supplement: S2 Fig — The two blue asterisks indicate the two image stacks that have particularly high total volume, total surface area and total tumour-stroma contact in Fig 5. (TIFF) [file pone.0143798.s008.tiff]

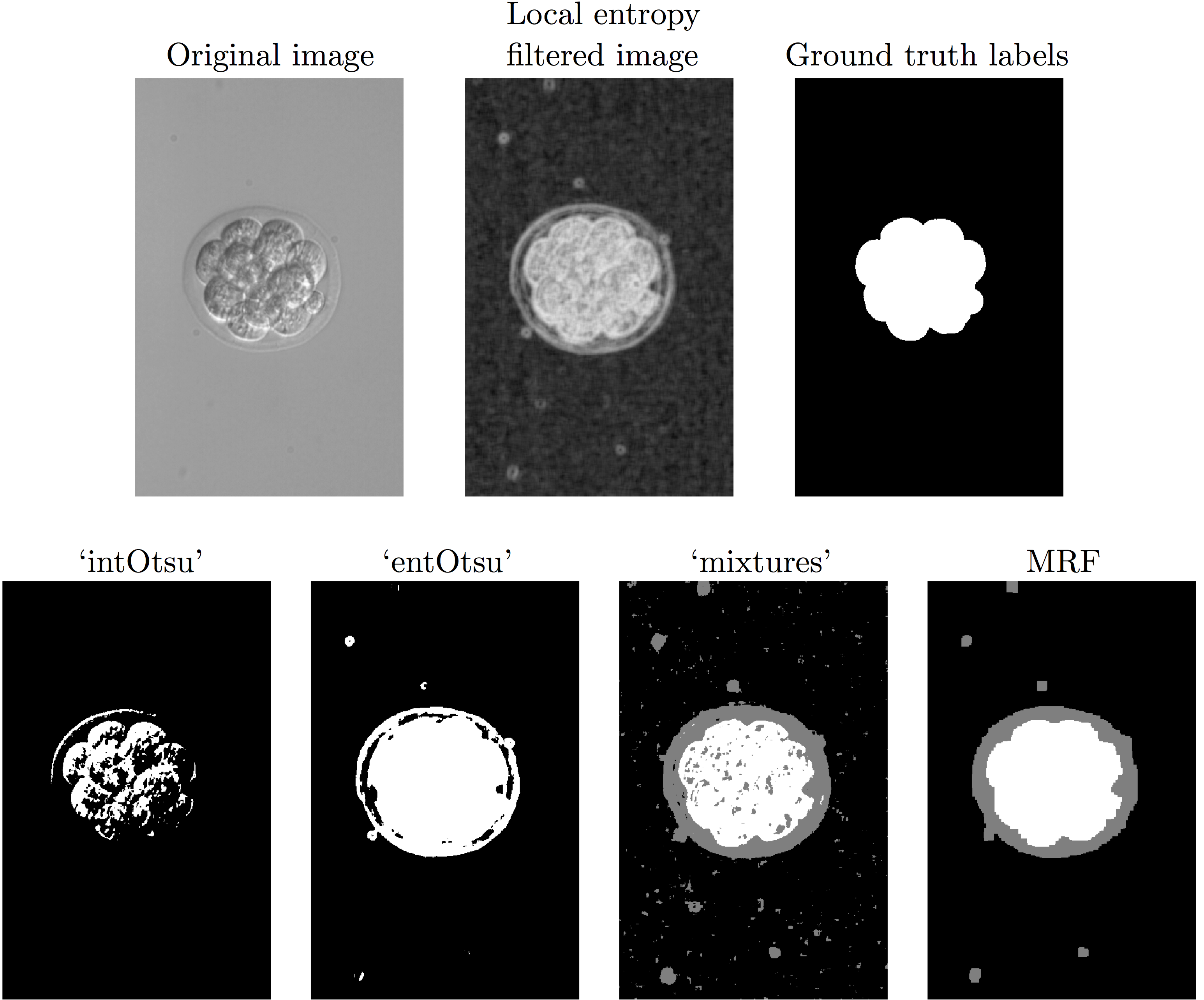

Supplement: S3 Fig — (TIFF) [file pone.0143798.s009.tiff]

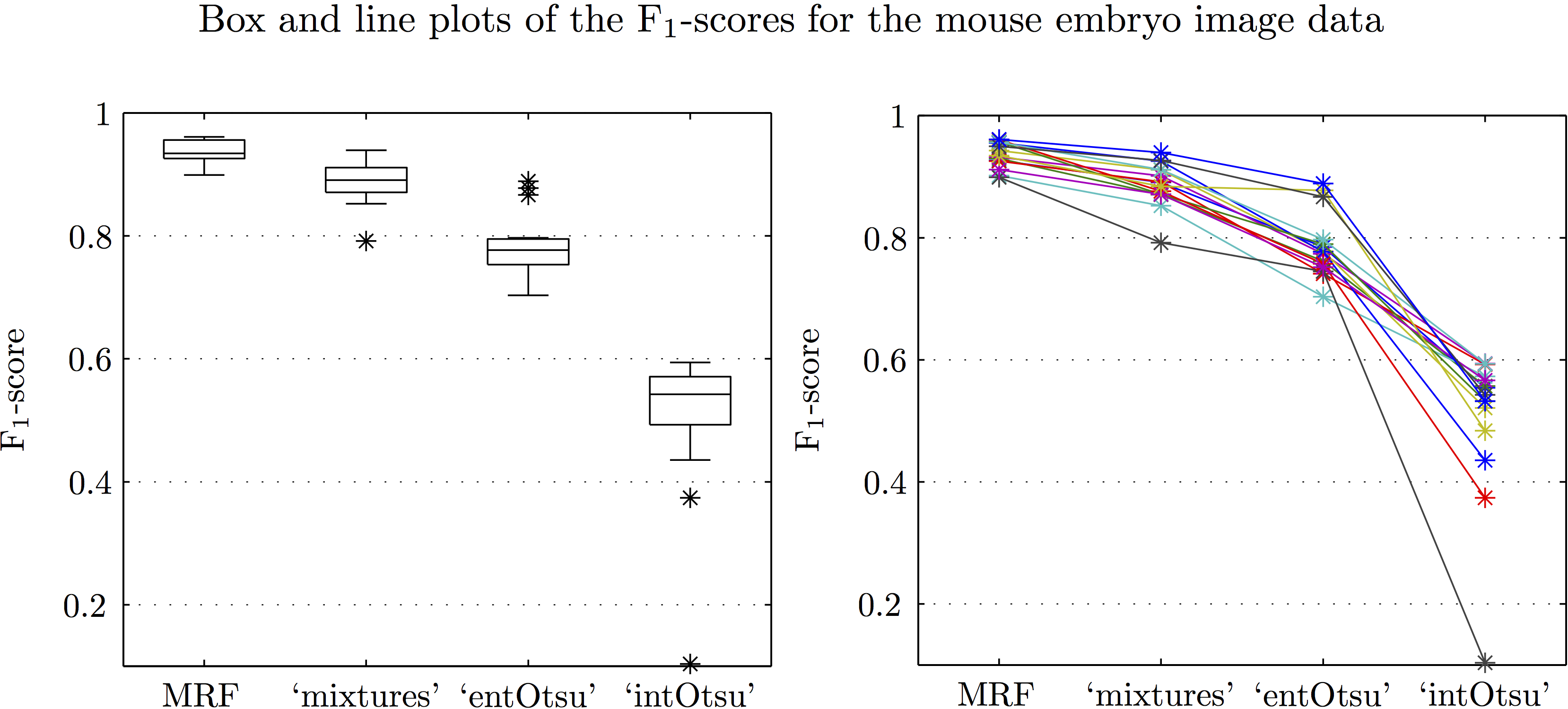

Supplement: S4 Fig — (TIFF) [file pone.0143798.s010.tiff]

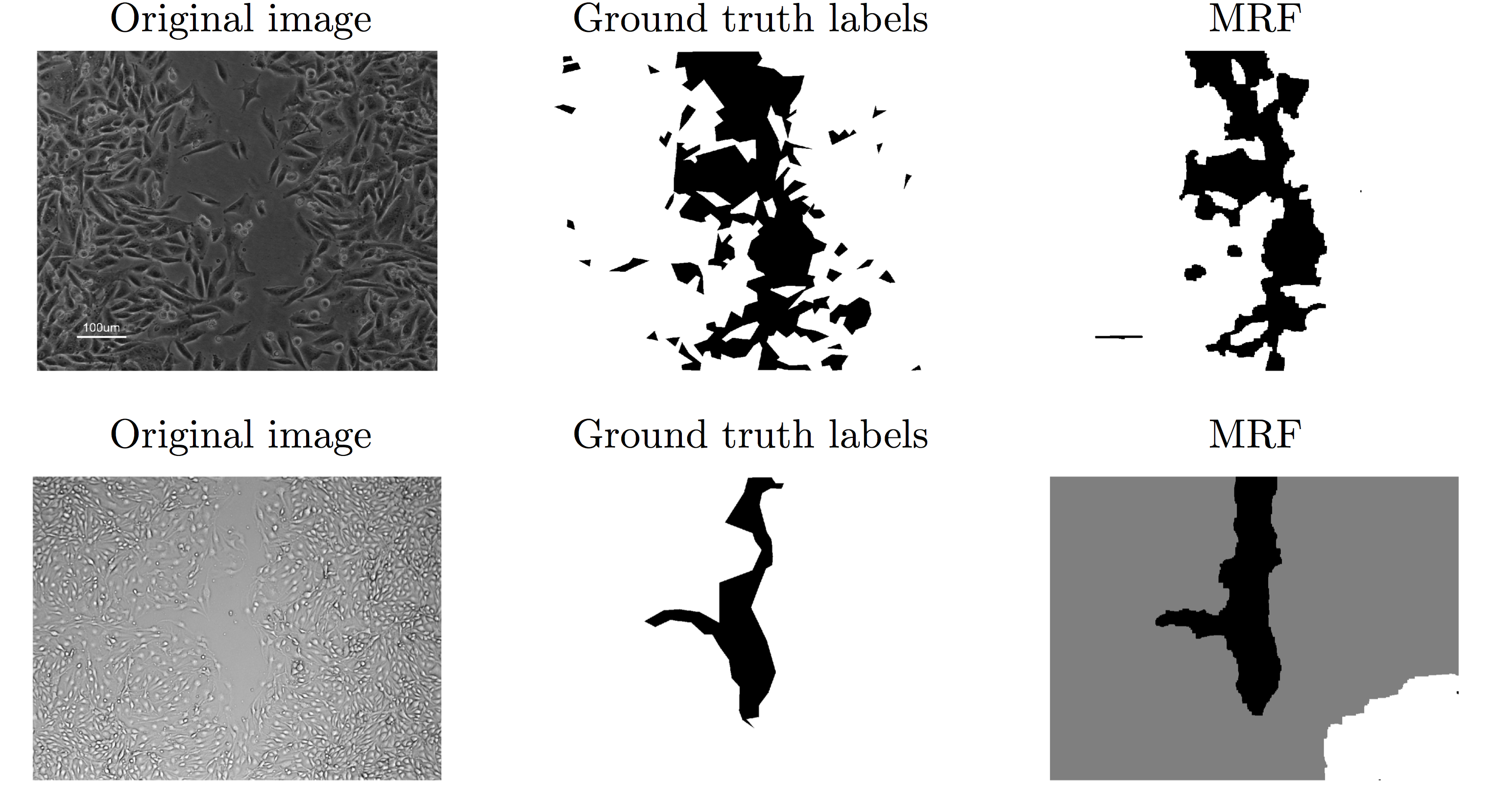

Supplement: S5 Fig — (TIFF) [file pone.0143798.s011.tiff]

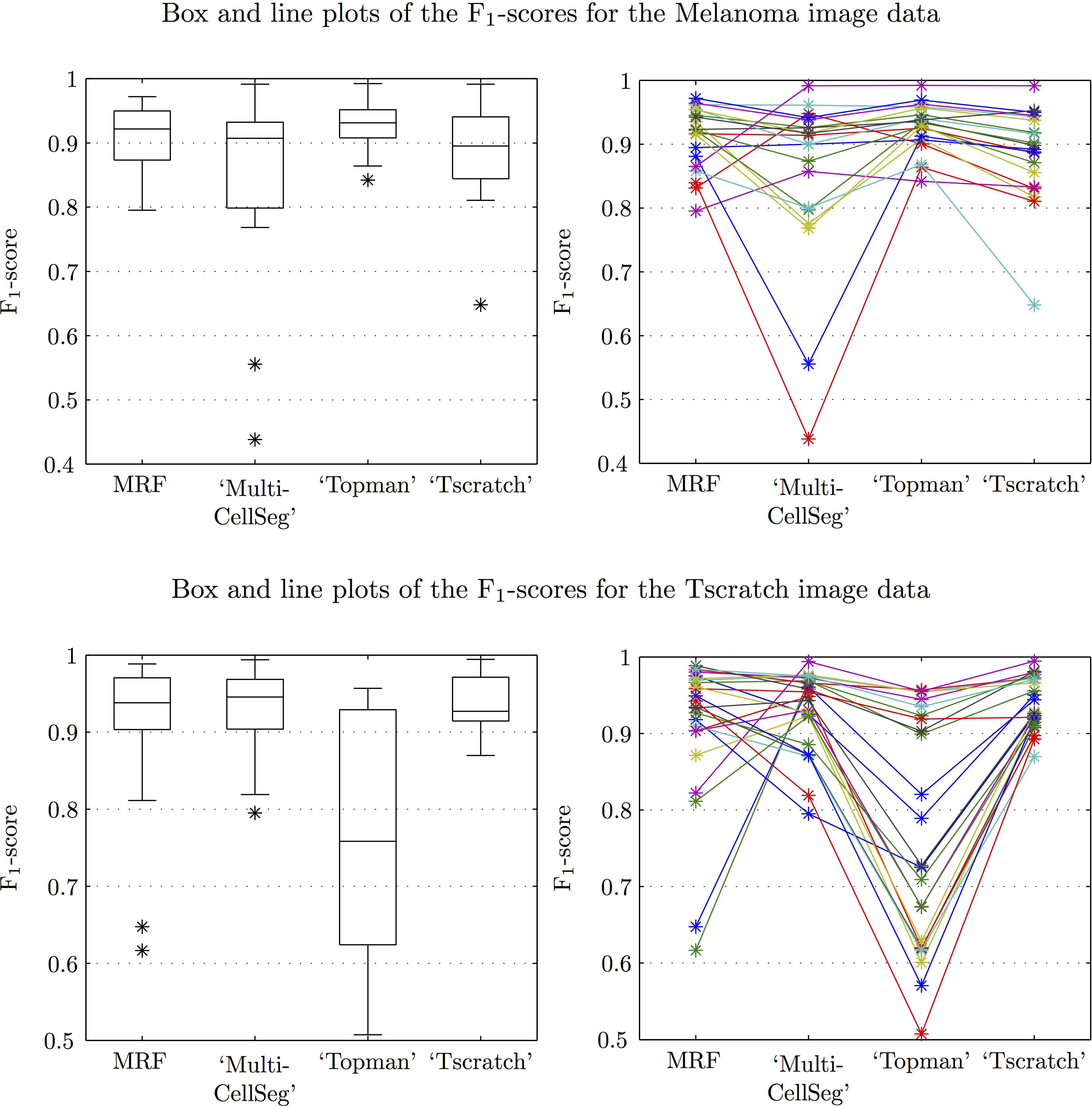

Supplement: S6 Fig — (TIFF) [file pone.0143798.s012.tiff]

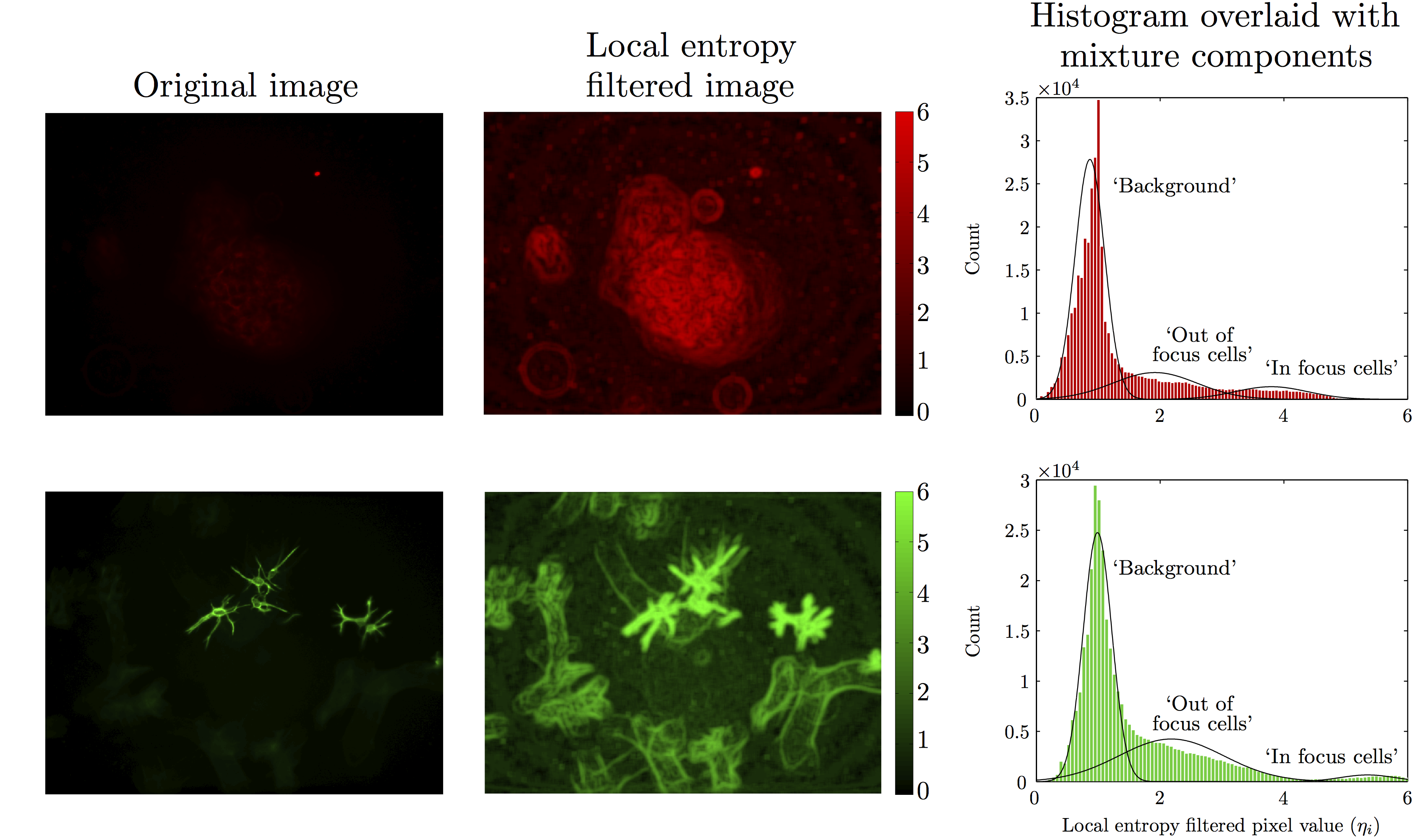

Supplement: S7 Fig — Example red and green channel images from a 3D stack with the corresponding local entropy filtered versions. The histograms of the local entropy filtered pixel values are overlaid with the three estimated mixture components. (TIFF) [file pone.0143798.s013.tiff]

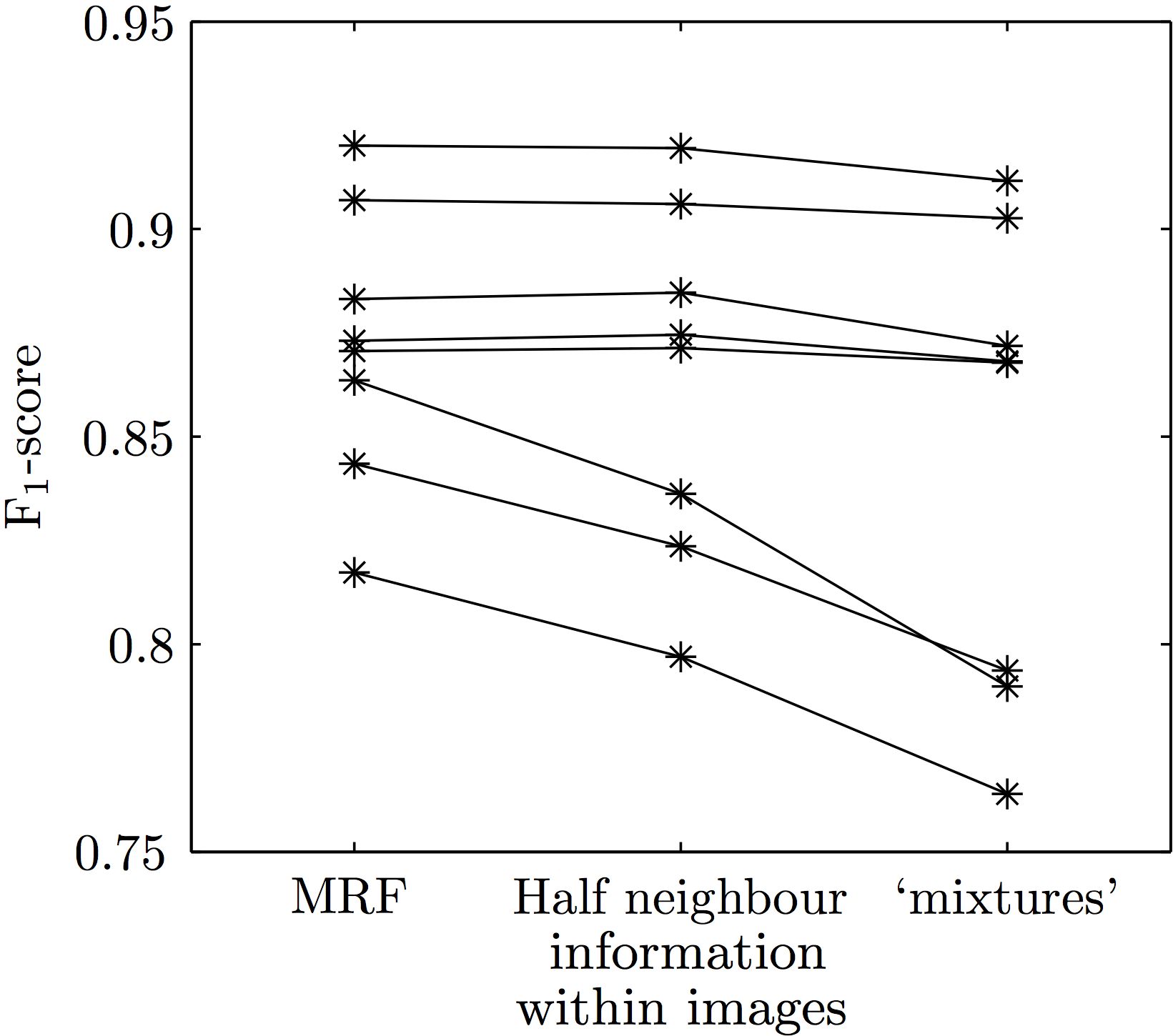

Supplement: S8 Fig — F1-scores for the MRF segmentation (negligible neighbour influence between images), the MRF segmentation with only the horizontal neighbours within images (a similar result is obtained using just the vertical neighbours) and the ‘mixtures’ segmentation (no neighbour influence at all). (TIFF) [file pone.0143798.s014.tiff]
