# Supplementary material for: Segmentation of Image Data from Complex Organotypic 3D Models of Cancer Tissues with Markov Random Fields
Source: PLoS One. 2015 Dec 2;10(12):e0143798. doi: 10.1371/journal.pone.0143798 (PMC4668034; doi:10.1371/journal.pone.0143798)
Supplement: S1 Table — (PDF) [file pone.0143798.s015.pdf]

	MRF	‘mixtures’	‘entOtsu’	‘intOtsu’
Mouse embryo image data	0.94 (0.93)	0.89 (0.89)	0.79 (0.78)	0.50 (0.54)
