# Supplementary material for: Segmentation of Image Data from Complex Organotypic 3D Models of Cancer Tissues with Markov Random Fields
Source: PLoS One. 2015 Dec 2;10(12):e0143798. doi: 10.1371/journal.pone.0143798 (PMC4668034; doi:10.1371/journal.pone.0143798)
Supplement: S2 Table — (PDF) [file pone.0143798.s016.pdf]

	MRF	‘MultiCellSeg’	‘Topman’	‘Tscratch’
Melanoma image data	0.91 (0.92)	0.85 (0.91)	0.93 (0.93)	0.88 (0.90)
Tscratch image data	0.91 (0.94)	0.93 (0.95)	0.78 (0.76)	0.94 (0.93)
